# Relative fat mass (RFM) as a new estimator of whole-body fat percentage ─ A cross-sectional study in American adult individuals

**DOI:** 10.1038/s41598-018-29362-1

**Published:** 2018-07-20

**Authors:** Orison O. Woolcott, Richard N. Bergman

**Affiliations:** Sports Spectacular Diabetes and Obesity Wellness and Research Center, Cedars-Sinai Medical Center, Los Angeles, CA 90048 USA

## Abstract

High whole-body fat percentage is independently associated with increased mortality. We aimed to identify a simple anthropometric linear equation that is more accurate than the body mass index (BMI) to estimate whole-body fat percentage among adult individuals. National Health and Nutrition Examination Survey (NHANES) 1999–2004 data (n = 12,581) were used for model development and NHANES 2005–2006 data (n = 3,456) were used for model validation. From the 365 anthropometric indices generated, the final selected equation was as follows: 64 − (20 × height/waist circumference) + (12 × sex), named as the relative fat mass (RFM); sex = 0 for men and 1 for women. In the validation dataset, compared with BMI, RFM better predicted whole-body fat percentage, measured by dual energy X-ray absorptiometry (DXA), among women and men. RFM showed better accuracy than the BMI and had fewer false negative cases of body fat-defined obesity among women and men. RFM reduced total obesity misclassification among all women and all men and, overall, among Mexican-Americans, European-Americans and African-Americans. In the population studied, the suggested RFM was more accurate than BMI to estimate whole-body fat percentage among women and men and improved body fat-defined obesity misclassification among American adult individuals of Mexican, European or African ethnicity.

## Introduction

High body fat percentage (adipose tissue mass relative to total body weight) is associated with mortality^1,2^. Recently, a large cohort study in adult individuals with a follow-up of 14 years reported that low baseline body mass index (BMI, weight in kilograms divided by the square of the height in meters) and high body fat percentage are independently associated with increased mortality^3^.Thus, accurate estimation of body fat percentage is highly relevant from a clinical and public health perspective, an aspect that has been endorsed by the American Heart Association Obesity Committee^4^.

Obesity, a state of excessive accumulation of body fat, is an important risk factor for multiple chronic pathologies including diabetes, coronary artery disease, hypertension and certain types of cancer^5–7^. Interestingly, the definition of obesity has changed over the last century. For example, early reports have defined obesity as the 20% to 40% excess of weight over the normal of 300 grams per centimeter of height^8^. Others have arbitrarily proposed body fat-defined obesity as a body fat percentage >35% for women and >25% for men^9^. To date, there is no consensus for the definition of obesity based on body fat percentage^10,11^. A BMI ≥30 is currently used to define obesity^12^. In fact, BMI is widely used to assess body fatness^12,13^, despite its limited accuracy to estimate body fat percentage^9,14,15^. An inherent problem of BMI due to its limited accuracy to estimate body fat percentage is misclassification of body fat-defined obesity. For example, a BMI ≥30 would overlook nearly 50% of women who had a body fat percentage higher than 35%^9^. Among the participants of the Third National Health and Nutrition Examination Survey, the diagnostic accuracy of BMI for body fat-defined obesity was estimated at 94% among women compared with 82% among men^9^. Thus, simple and low-cost alternatives to BMI with better diagnostic accuracy for obesity in both sexes would be of considerable importance.

Although several sophisticated techniques are available to obtain accurate estimates of whole-body fat percentage^16^; these methods are unsuitable for routine clinical purposes and large population studies. Consequently, numerous equations based on anthropometrics have been proposed as alternatives to BMI to better estimate whole-body fat percentage^17–25^. Some published equations require more than 10 different anthropometric measurements^19^, others require up to four different skinfold measurements^21^; some are relatively complex equations with numerous terms^20,25^. Thus, one common problem among existing equations is the lack of simplicity, showing limited potential for their use in routine clinical practice or public health.

In the present study, we systematically explored more than 350 anthropometric indices aiming to identify a simple anthropometric linear equation that is more accurate than the BMI as a potential alternative tool for clinical and epidemiological purposes to estimate whole-body fat percentage among female and male adult individuals. The second aim of the study was to evaluate its clinical utility.

## Results

### Study population

**Figure 1:**
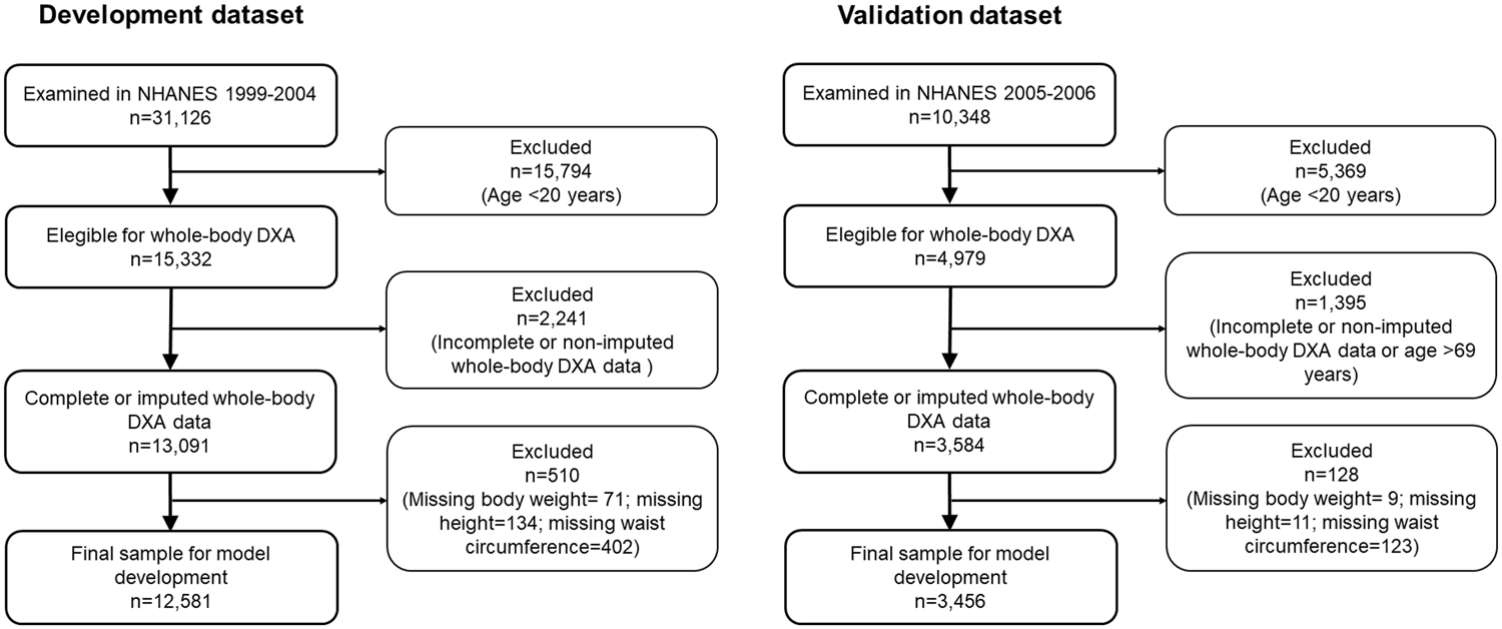
Flow diagram of participant selection for the development and validation datasets. DXA, dual energy X-ray absorptiometry.

We included for analysis data from adult individuals 20 years of age and older who participated in the National Health and Nutrition Examination Survey (NHANES) 1999–2006. NHANES 1999–2004 data (n = 12,581) were used for model development and NHANES 2005–2006 data (n = 3,456) were used for model validation. Participants selection for the development and validation datasets is shown in Fig. 1. Characteristics of the participants studied are described in Table 1. Mean values of whole-body fat percentage measured by dual energy X-ray absorptiometry (DXA) in the development and validation datasets were 39.9% and 39.4% in women, and 28.0% and 27.8% in men, respectively. The frequencies of DXA multiply imputed data in the development and validation datasets are described in Supplementary Tables 1 and 2, respectively.

**Table 1:** Characteristics of adult individuals (≥20 years old) included in the study^*^.

	NHANES 1999–2004		NHANES 2005–2006		P value	
	(Development dataset)		(Validation dataset)			
	Women	Men	Women	Men	For Women	For Men
	N = 6,261 (51%)	N = 6,320 (49%)	N = 1,700 (50.3%)	N = 1,756 (49.7%)		
Age, yr	47.2 ± 0.3	45.0 ± 0.3	43.3 ± 0.4	42.1 ± 0.6	<0.001	<0.001
**Ethnicity**					0.23	0.54
*Mexican-American, %*	6.4 ± 0.9	8.0 ± 0.9	7.3 ± 0.9	9.5 ± 1.3		
European-American*, %*	71.7 ± 1.8	72.2 ± 1.6	69.7 ± 3.1	71.2 ± 2.8		
African-American*, %*	11.3 ± 1.1	9.9 ± 0.9	12.4 ± 2.2	10.8 ± 1.7		
**Age category**					<0.001	<0.001
*20–39 years old, %*	36.6 ± 1.0	41.3 ± 1.0	39.7 ± 1.3	44.1 ± 1.9		
*40–59 years old, %*	39.1 ± 0.9	39.1 ± 0.7	46.9 ± 1.4	43.9 ± 1.4		
≥*60 years old, %*	24.2 ± 0.7	19.7 ± 0.6	13.4 ± 1.1	12.1 ± 1.3		
**BMI category**					0.10	0.02
<*18.5, %*	2.5 ± 0.3	1.2 ± 0.2	2.2 ± 0.5	1.2 ± 0.3		
*18.5–24.9, %*	35.8 ± 1.1	30.0 ± 0.7	36.6 ± 1.9	25.4 ± 1.8		
*25–29.9, %*	28.8 ± 0.9	40.9 ± 0.8	24.8 ± 1.2	39.6 ± 1.4		
≥*30, %*	32.9 ± 1.0	28.0 ± 0.7	36.4 ± 1.7	33.8 ± 2.3		
**Anthropometry**						
Body weight, kg	74.1 ± 0.4	86.8 ± 0.3	75.8 ± 0.9	89.5 ± 0.9	0.08	0.005
Height, cm	162.2 ± 0.1	176.2 ± 0.1	162.6 ± 0.2	176.6 ± 0.2	0.05	0.08
BMI, kg/m^2^	28.2 ± 0.1	27.9 ± 0.1	28.7 ± 0.3	28.6 ± 0.3	0.17	0.01
Waist circumference, cm	93.1 ± 0.4	99.5 ± 0.3	93.9 ± 0.8	100.8 ± 0.8	0.37	0.12
Whole-body fat mass, kg	30.8 ± 0.3	25.3 ± 0.2	31.2 ± 0.6	26.0 ± 0.5	0.47	0.23
Whole-body fat free mass, kg	41.9 ± 0.2	59.5 ± 0.2	43.1 ± 0.3	61.6 ± 0.4	0.002	<0.001
Whole-body fat percentage	39.9 ± 0.2	28.0 ± 0.1	39.4 ± 0.3	27.8 ± 0.3	0.17	0.48
Trunk fat percentage	38.2 ± 0.2	29.1 ± 0.1	37.5 ± 0.3	28.8 ± 0.4	0.13	0.38

^*^Values represent pooled weighted mean estimates (or percentages, as indicated) ± standard errors. Percentages may not total 100 due to rounding. BMI, body mass index (weight in kilograms divided by the square of the height in meters). P values were calculated using the Wald test.

### Model development, performance, and selection

Supplementary Table 3 shows correlation matrix among the commonly used anthropometrics including body weight, height, BMI, triceps and subscapular skinfolds, arm and leg lengths, and waist, calf, arm and thigh circumferences. Since arm and leg lengths showed poor correlation with body fat percentage, they were excluded from further analysis. In total, 365 anthropometric indices were empirically generated and tested for correlation with body fat percentage (see Supplementary Table 4 for a full list of all indices generated).

Equations were derived using linear regression. Our selected regression models included those based on the simplest indices with the highest correlation with body fat percentage among women and among men. Among the 365 generated indices, height^3^/(waist × weight) showed the highest correlation with whole-body fat percentage among women (r = −0.81; P < 0.001). (√Height)/waist equation showed the highest correlation with whole-body fat percentage among men (r = −0.85; P < 0.001). Height^3^/(waist × weight) showed slightly stronger correlation than the simple 1/BMI (r = −0.79; P < 0.001) among women. Among men, (√height)/waist showed slightly stronger correlation than the simpler index height/waist (r = −0.84; P < 0.001). Height^2^/(waist × √weight) showed high correlations both among women and men. Thus, we finally selected the five aforementioned indices to evaluate model performance.

Given height/waist is the reciprocal of the widely used waist-to-height ratio, we also examined the predicting ability of waist/height index. Height/waist better predicted whole-body fat percentage and showed lower root mean squared error (RMSE) than waist/height among men and women, and across ethnic groups (Supplementary Table 5) and age categories (Supplementary Table 6). Thus, we dropped waist/height from further analysis. Supplementary Fig. 1 shows improved linear relationship between whole-body fat percentage and height/waist by sex and ethnicity. All selected models showed lower prediction of body fat percentage in older individuals (Supplementary Table 6). We found a progressive decline in body weight, height and fat-free mass after 50 years of age, and a steeper decline in fat mass and waist circumference after 70 years of age among women and men (Supplementary Fig. 5), which coincided with the lower predicting ability of all models in older individuals.

For practical reasons, performance analyses of all selected models presented here were tested using their rounded and simplest expression (details are provided in the Supplementary material). Raw equations are shown in Supplementary Table 7. Concordance coefficients between DXA-measured whole-body fat percentage and final selected models are shown in Supplementary Table 8.

All selected models showed higher accuracy than BMI among women, whereas precision was improved only in models based on three anthropometrics and 1/BMI (Supplementary Table 9). Among men, height/waist equation showed the highest accuracy, and was also superior to BMI. Models based on three anthropometrics but not 1/BMI were also more accurate than BMI. All models but not 1/BMI were more precise than BMI among men (Supplementary Table 9).

Height/waist equation, named as the relative fat mass (RFM), was the final model selected because of its simplicity (it requires only two common anthropometrics), it was superior to BMI in predicting body fat percentage among men, had similar predicting ability relative to BMI among women and had overall better performance than BMI among women and men, independently.

Final equations are as follows:

1 $${\rm{Equation}}\,{\rm{for}}\,{\rm{women}}:\,76-(20\times ({\rm{height}}/{\rm{waist}}))$$

2 $${\rm{Equation}}\,{\rm{for}}\,{\rm{men}}:\,64-(20\times ({\rm{height}}/{\rm{waist}}))$$

or

3 $${\rm{RFM}}:\,64-(20\times ({\rm{height}}/{\rm{waist}}))+(12\times {\rm{sex}})$$

In equations (1–3), height and waist (circumference) are expressed in meters. In (3), sex = 0 for male and 1 for female. The coefficients for equations (1) and (2) were rounded for practical purposes.

Supplementary Fig. 3 shows good agreement between RFM and DXA.

Although we found a significant interaction between age and RFM among women (P < 0.001), that was not case among men (P = 0.088). However, inclusion of age in the final model did not improve R^2^ among women (RFM model: R^2^ = 0.66; RFM and AGE model: R^2^ = 0.66) or among men (RFM model: R^2^ = 0.75; RFM and AGE model: R^2^ = 0.75). Likewise, inclusion of ethnicity in the final model did not substantially increased R^2^ among men (RFM and ethnicity model: R^2^ = 0.76). Among women, inclusion of ethnicity in the model did not improve body fat prediction (R^2^ = 0.66). Thus, age and ethnicity were not included in our final model selected.

### Model validation and performance

**Figure 2:**
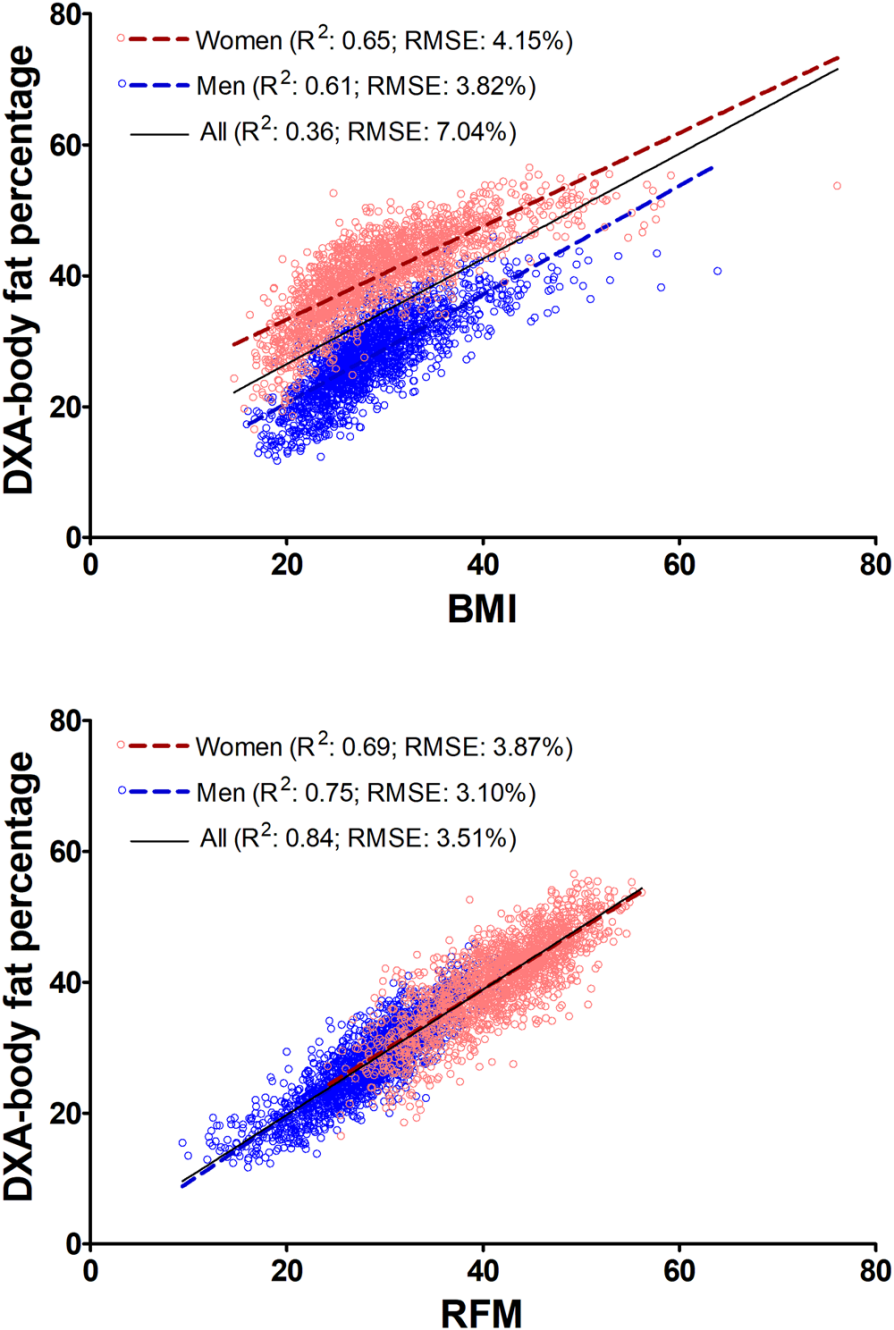
Prediction of whole-body fat percentage by RFM using linear regression in NHANES 2005–2006 (validation dataset). RFM, relative fat mass, which is based on height/waist. R^2^, coefficient of determination; RMSE, root mean squared error. Data plots correspond to DXA imputation 1.

In the validation dataset, compared with BMI, RFM had a more linear relationship with DXA whole-body fat percentage among women (adjusted coefficient of determination, R^2^: 0.69; 95% CI, 0.67–0.72; vs. 0.65; 95% CI, 0.63–0.67) and men (R^2^: 0.75; 95% CI, 0.72–0.77 vs. 0.61; 95% CI, 0.59–0.63) (Fig. 2 and Supplementary Table 10). RFM had less bias than BMI among women (0.9%; 95% CI, 0.6% to 1.1% vs. −10.9%; 95% CI, −11.2% to −10.5%) and a similar low bias among men (RFM: 0.5%; BMI: 0.7%) (Table 2). Among women, RFM showed higher accuracy than BMI (91.5% vs. 21.6%; P < 0.001). RFM was also more precise than BMI (4.9%; 95% CI, 4.6–5.2% vs. 5.8%; 95% CI, 5.5–6.2%). Among men, RFM showed higher accuracy than BMI (88.9% vs. 81.9%; P < 0.001) and better precision (RFM: 4.2%; 95% CI, 3.9–4.6% vs. BMI: 5.1%; 95% CI, 4.9–5.4%) (Table 2 and Supplementary Fig. 4). Among women, RFM was more accurate than BMI across ethnic groups (P < 0.001 for all comparisons). Among men, RFM was also more accurate among European-Americans (P < 0.001) and African-Americans (P < 0.001) (Table 2). RFM also showed better performance than BMI across age categories (Supplementary Fig. 5) and across body fat quintiles (Supplementary Fig. 6). Among men, RFM also showed better performance than CUN-BAE (Clinica Universidad de Navarra-body adiposity estimator), Gallagher, Deurenberg and Kagawa equations, including across ethnic groups. Among women, RFM was superior to Deurenberg and Kagawa equations (Table 2).

**Table 2:** Comparison of performance between RFM and published equations based on BMI or waist-to-height ratio for prediction of body fat percentage among adult participants (n = 3,456) in the validation dataset (NHANES 2005–2006)*.

	All	Mexican-American	European-American	African-American
**Women (n = 1,700)**				
**Bias (95% CI)**				
*BMI*	−10.9 (−11.2 to −10.5)	−11.0 (−11.5 to −10.5)	−11.0 (−11.3 to −10.6)	−9.3 (−10.0 to −8.6)
*RFM* ^†^	0.9 (0.6 to 1.1)	1.5 (1.1 to 2.0)	0.6 (0.4 to 0.9)	1.5 (0.6 to 2.3)
*CUN-BAE equation* ^‡^	−0.2 (−0.5 to 0.1)	−0.4 (–1.4 to 0.7)	−0.4 (−0.7 to 0.0)	1.6 (0.9 to 2.4)
*Gallagher equation* ^§^	−2.8 (−3.1 to −2.6)	−2.9 (−3.9 to −2.0)	−2.9 (−3.1 to −2.6)	−1.4 (−2.2 to −0.6)
*Deurenberg equation* ^*¶*^	−2.3 (−2.7 to −2.0)	−2.9 (−3.8 to −2.1)	−2.3 (−2.7 to −1.8)	−0.3 (−1.2 to 0.6)
*Kagawa equation* ^#^	1.9 (1.5 to 2.4)	3.6 (2.7 to 4.5)	1.6 (1.2 to 2.0)	3.9 (3.0 to 4.8)
**Accuracy (95% CI)**				
*BMI*	21.6 (18.9 to 24.4)	17.8 (13.4 to 22.2)	20.4 (17.5 to 23.3)	36.3 (30.8 to 41.8)
*RFM*	91.5 (89.9 to 93.0)	91.7 (88.2 to 95.3)	92.1 (90.1 to 94.2)	89.4 (86.2 to 92.5)
*CUN-BAE equation*	92.0 (90.3 to 93.7)	91.0 (86.7 to 95.2)	93.0 (90.9 to 95.2)	92.1 (88.4 to 95.9)
*Gallagher equation*	88.4 (86.1 to 90.8)	87.5 (82.5 to 92.4)	88.9 (85.7 to 92.1)	91.8 (88.2 to 95.4
*Deurenberg equation*	79.0 (76.9 to 81.1)	76.5 (69.3 to 83.6)	80.4 (77.8 to 83.0)	79.8 (75.7 to 83.8)
*Kagawa equation*	82.8 (80.9 to 84.8)	76.1 (69.7 to 82.6)	84.8 (82.5 to 87.1)	75.1 (70.7 to 79.5)
**Precision (95% CI)**				
*BMI*	5.8 (5.5 to 6.2)	4.7 (4.0 to 5.4)	5.7 (5.3 to 6.1)	5.7 (5.1 to 6.2)
*RFM*	4.9 (4.6 to 5.2)	4.6 (4.0 to 5.3)	4.9 (4.5 to 5.2)	5.3 (4.7 to 5.9)
*CUN-BAE equation*	6.0 (5.7 to 6.3)	6.0 (5.4 to 6.5)	5.9 (5.5 to 6.3)	5.6 (4.8 to 6.5)
*Gallagher equation*	5.2 (4.9 to 5.5)	5.0 (4.4 to 5.7)	5.1 (4.7 to 5.5)	5.1 (4.5 to 5.7)
*Deurenberg equation*	7.5 (7.1 to 8.0)	7.5 (6.6 to 8.5)	7.3 (6.6 to 7.9)	8.3 (7.5 to 9.1)
*Kagawa equation*	7.3 (6.9 to 7.7)	7.2 (6.2 to 8.2)	7.2 (6.8 to 7.7)	7.7 (6.8 to 8.6)
**Men (n = 1,756)**				
**Bias (95% CI)**				
*BMI*	0.7 (0.5 to 0.9)	0.6 (0.1 to 1.0)	0.5 (0.3 to 0.8)	2.8 (2.1 to 3.5)
*RFM*	0.5 (0.3 to 0.8)	1.0 (0.5 to 1.5)	0.5 (0.2 to 0.7)	0.9 (0.3 to 1.4)
*CUN-BAE equation*	−0.1 (−0.4 to 0.2)	−0.9 (−1.4 to −0.33)	−0.24 (−0.7 to 0.2)	2.0 (1.3 to 2.7)
*Gallagher equation*	−3.7 (−3.8 to −3.5)	−4.4 (−4.8 to −4.1)	−3.7 (−3.9 to −3.5)	−1.7 (−2.3 to −1.1)
*Deurenberg equation*	−1.9 (−2.3 to −1.6)	−3.7 (−4.2 to −3.1)	−1.9 (−2.4 to −1.4)	−0.3 (−1.3 to 0.8)
*Kagawa equation*	2.3 (2.0 to 2.6)	3.1 (2.9 to 3.3)	2.1 (1.8 to 2.5)	2.3 (1.5 to 3.2)
**Accuracy (95% CI)**				
*BMI*	81.9 (79.6 to 84.3)	88.9 (87.0 to 90.7)	82.6 (79.7 to 85.4)	67.1 (57.8 to 76.5)
*RFM*	88.9 (86.8 to 91.1)	91.3 (88.9 to 93.7)	88.7 (86.0 to 91.3)	86.7 (81.3 to 92.1)
*CUN-BAE equation*	79.1 (76.6 to 81.7)	83.3 (78.6 to 88.1)	79.7 (76.9 to 82.6)	70.0 (61.1 to 78.8)
*Gallagher equation*	71.0 (68.4 to 73.7)	64.6 (58.5 to 70.6)	71.2 (67.7 to 74.7)	80.4 (76.8 to 84.0)
*Deurenberg equation*	69.4 (66.8 to 72.0)	64.4 (58.8 to 69.9)	69.8 (66.4 to 73.2)	71.9 (67.2 to 76.5)
*Kagawa equation*	76.1 (73.4 to 78.8)	74.8 (69.0 to 80.5)	76.3 (73.4 to 79.3)	71.5 (64.6 to 78.3)
**Precision (95% CI)**				
*BMI*	5.1 (4.9 to 5.4)	4.1 (3.6 to 4.7)	5.2 (4.8 to 5.6)	5.2 (4.5 to 5.8)
*RFM*	4.2 (3.9 to 4.6)	3.8 (3.3 to 4.3)	4.4 (4.0 to 4.8)	3.9 (3.5 to 4.3)
*CUN-BAE equation*	5.7 (5.3 to 6.2)	5.3 (4.6 to 5.9)	5.8 (5.2 to 6.3)	5.7 (5.1 to 6.4)
*Gallagher equation*	5.0 (4.4 to 5.5)	4.3 (3.8 to 4.8)	5.0 (4.4 to 5.6)	4.9 (4.3 to 5.5)
*Deurenberg equation*	6.2 (5.8 to 6.6)	5.6 (4.7 to 6.5)	6.1 (5.5 to 6.8)	5.7 (5.0 to 6.5)
*Kagawa equation*	5.0 (4.6 to 5.4)	4.1 (3.5 to 4.7)	5.1 (4.7 to 5.5)	5.3 (4.7 to 5.8)

^*^Values represent weighted estimates with 95% confidence intervals (95% CI) from DXA imputed data. Model performance was evaluated as follows: Bias was calculated as the median difference between estimated and measured body fat percentage. Accuracy was calculated as the proportion of cases with <20% difference between estimated and measured body fat percentage. Precision was calculated as the confidence interval of the interquartile range of the difference between estimated and measured body fat percentage.

^†^RFM equation: 64 − (20 × height/waist) + (12 × sex).

^‡^CUN-BAE equation: −44.988 + (0.503 × age) + (10.689 × sex) + (3.172 × BMI) − (0.026 × BMI^2^) + (0.181 × BMI × sex) − (0.02 × BMI × age) − (0.005 × BMI^2^ × sex) + (0.0002 × BMI^2^ × age).

^§^Gallagher equation: 64.5 − (848 × (1/BMI)) + (0.079 × age) − (16.4 × sex) + (0.05 × sex × age) + (39.0 × sex × (1/BMI)).

^¶^Deurenberg equation: − (11.4 × sex) + (0.20 × age) + (1.294 × BMI) − 8.

^#^Kagawa equation: − 8.339 + (92.701 × waist/height) − (0.078 × age) − (11.062 × sex).

For RFM and CUN-BAE equations, sex = 0 for male and 1 for female. For Gallagher, Deurenberg and Kagawa equations, sex = 1 for male and 0 for female.

For CUN-BAE, Gallagher, Deurenberg and Kagawa equations, age in years.

For RFM and Kagawa equations, height and waist (circumference) in meters.

BMI, body mass index (body weight in kilograms divided by squared height in meters); CUN-BAE, Clinica Universidad de Navarra-body adiposity estimator; RFM, relative fat mass.

Internal validation with bootstrapping confirmed RFM was a better predictor of body fat percentage than BMI among women and men (Supplementary Table 11). RFM predicting ability decreased with age (Supplementary Table 12). RFM was more accurate and more precise than BMI (Supplementary Table 13) and had superior accuracy than BMI across age categories (Supplementary Fig. 7 and Supplementary Table 14) and body fat ranges; however, accuracy was lower in leaner individuals (Supplementary Fig. 8).

RFM was a better predictor of trunk fat percentage than of whole-body fat percentage or whole-body fat mass (Supplementary Table 15).

### Obesity misclassification

To compare the rates of obesity misclassification between BMI and our final model, we arbitrarily defined obesity as DXA-measured body fat percentage ≥33.9% for women and ≥22.8% for men, based on the corresponding cut-points between the first and second quintiles for each sex. These cut-points were calculated using combined datasets (NHANES 1999–2006). In the validation dataset, when using same DXA cut-points for obesity diagnosis (≥33.9% for women and ≥22.8% for men), RFM had higher sensitivity than BMI. Table 3 shows total positive and negative cases of obesity identified using either BMI or RFM. RFM resulted in fewer false negatives among women (5.0%; 95% CI, 3.1–6.8% vs. 72.0%; 95% CI, 67.3–76.6%; P < 0.001) and men (3.8%; 95% CI, 1.8–5.8% vs. 4.1%; 95% CI, 2.1–6.1%; P < 0.001). There were fewer false positives with RFM among men (32.3%; 95% CI, 25.8–38.8% vs. 49.7%; 95% CI, 44.2–55.3%; P < 0.001) but more false positives among women (41.0%; 95% CI, 32.2–49.9% vs. 0%; P < 0.001).

**Table 3:** Positive and negative cases of DXA-diagnosed obesity* identified using either BMI or RFM among adult participants (n = 3,456) in the validation dataset (NHANES 2005–2006).

	DXA-Negative	DXA-Positive	Total		DXA-Negative	DXA-Positive	Total
**Women**							
BMI-Negative	622	1,960	2,582	RFM-Negative	362	110	472
BMI-Positive	0	818	818	RFM-Positive	260	2,668	2,928
Total	622	2,778	3,400	Total	622	2,778	3,400
**Men**							
BMI-Negative	366	70	436	RFM-Negative	468	74	542
BMI-Positive	386	2,690	3,076	RFM-Positive	284	2,686	2,970
Total	752	2,760	3,512	Total	752	2,760	3,512

^*^Obesity was defined as a DXA body fat percentage ≥33.9% for women and ≥22.8% for men based on the cut-points between the first and second quintiles for each sex. DXA, dual energy X-ray absorptiometry.

**Figure 3:**
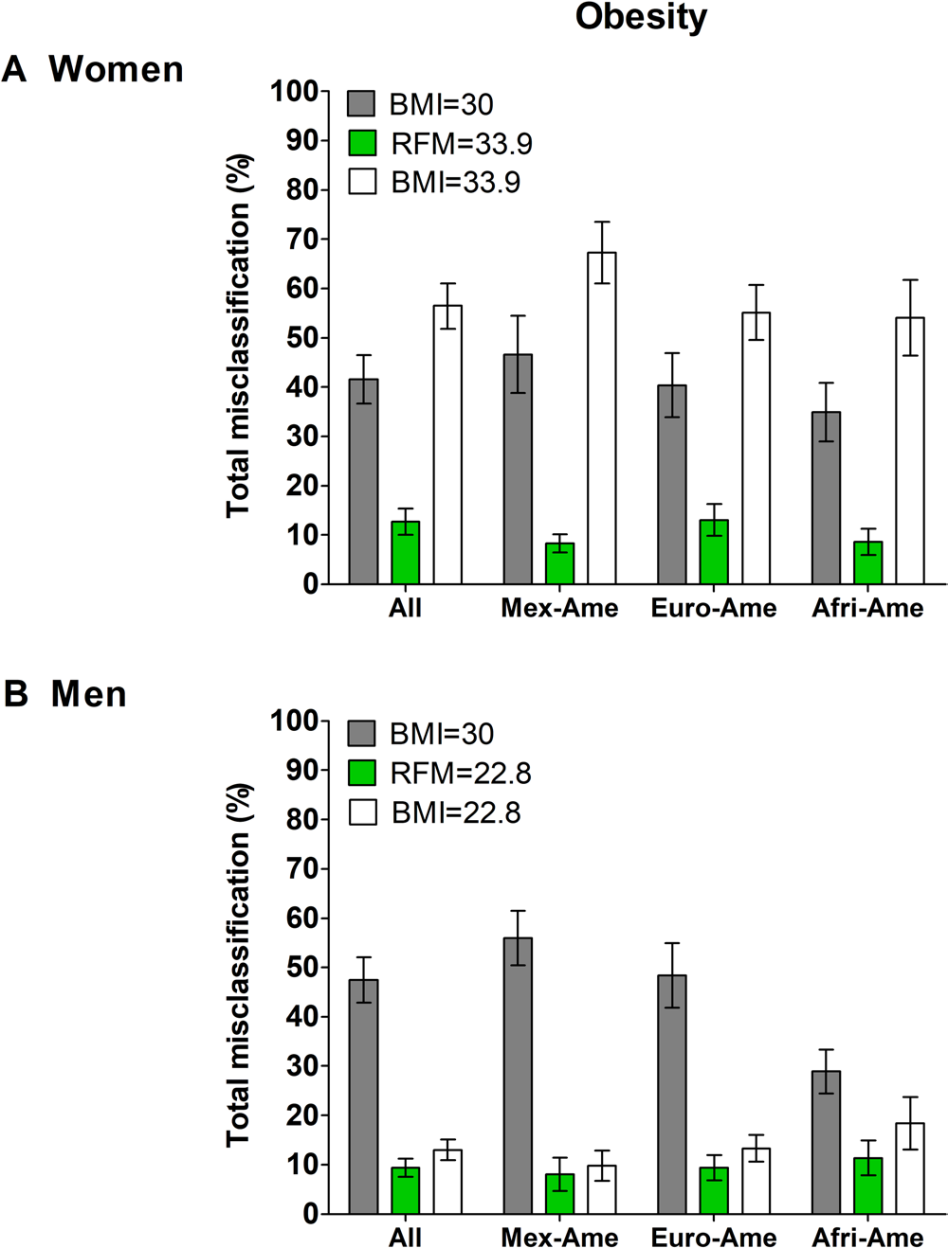
Obesity total misclassification error in NHANES 2005–2006. Bars show comparison of total misclassification of obesity diagnosed by DXA-whole-body fat percentage (≥33.9% for women and ≥22.8% for men, based on the corresponding cut-points between the first and second quintiles for each sex) when using RFM and BMI at same DXA cut-points and a BMI of 30. Error bars are standard error.

Obesity total misclassification was also lower with RFM than with BMI among all women (12.7% vs. 56.5%; P < 0.001) and all men (9.4% vs. 13.0%; P < 0.001) (Fig. 3), and among all Mexican-Americans (8.2% vs. 35.4%; P < 0.001), all European-Americans (11.3% vs. 35.2%; P < 0.001) and all African-Americans (9.9% vs. 37.2%; P < 0.001).

In the internal validation dataset, compared with BMI, obesity total misclassification was lower with RFM among women (P < 0.001) and men (P < 0.001), among all Mexican-Americans, all European-Americans and all African-Americans (P < 0.001 for all three ethnic groups), and across age categories (P < 0.001 for all comparisons). Although we found a lower total misclassification rate with RFM among other ethnicities (Non-Hispanic Asians, Native Americans, and those who self-reported multiple ethnicity) (RFM: 12.9%, BMI: 41.9%; P < 0.001), these findings should be interpreted with caution as NHANES 1999–2006 did not oversample to get reliable estimates on these minority American ethnic groups.

### Diagnostic accuracy for obesity and diabetes

In the validation dataset, compared with BMI, RFM showed better diagnostic accuracy for body fat-defined obesity among men (area under curve [AUC]: 0.94 vs. 0.91; P < 0.001) and similar diagnostic accuracy among women (AUC: 0.929 vs. 0.933; P = 0.52). RFM was also better than BMI in identifying diabetes cases among women (AUC: 0.79 vs 0.73; P = 0.002) and men (AUC: 0.80 vs. 0.76; P = 0.001).

Sensitivity analysis of the combined datasets showed RFM had a better diagnostic accuracy than BMI for high body-fat percentage among men (P < 0.001) regardless the DXA cut-point used to define obesity (Supplementary Fig. 9). RFM also showed a significant improvement over BMI and Gallagher, CUN-BAE and Deurenberg equations among men (Supplementary Table 16).

RFM was superior to DXA-measured trunk fat percentage in discriminating diabetes among women (P < 0.001) but not among men (P = 0.548) (Supplementary Fig. 10).

## Discussion

In the present study, we identified the relative fat mass (RFM), which is a simple linear equation based on height-to-waist ratio, as a potential alternative tool to estimate whole-body fat percentage in women and men 20 years of age and older. Our analyses were performed using nationally representative samples of the US adult population which allowed us to evaluate the performance of RFM among Mexican-Americans, European Americans, and Africans-Americans.

In the validation dataset, the performance of RFM to estimate DXA-measured body fat percentage was overall more consistent than that of BMI among women and men, across ethnic groups, young, middle-age and older adults, and across quintiles of body fat percentage, although the accuracy of RFM was lower among individuals with lower body fatness. RFM also showed overall better performance (accuracy and precision) than the CUN-BAE, Gallagher, Deurenberg and Kagawa equations to estimate whole-body fat percentage among women and men.

The selection of our final model deserves some comment. The main aim of the present study was to identify a simple anthropometric equation, that could potentially be used for clinical and epidemiological purposes, as an alternative to BMI to better assess body fatness among adult individuals. No attempt was made to generate non-linear equations or complex linear equations based on a high number of anthropometrics. Previous studies have addressed this point^19,22^. Although our selected models based on three anthropometrics showed the highest adjusted R-squared than those based on two anthropometrics among women, we believe they would unlikely represent a practical alternative to BMI. Although the equations based on 1/BMI and height/waist showed a good predicting value among women and men, respectively, a different index for each sex would also result in low practicality when compared with BMI. Thus, we finally selected the height/waist equation (RFM) because it was the simplest equation among all selected models that better estimated whole-body fat percentage than the BMI among women and men, independently. Although waist-to-height ratio is widely used in epidemiology as a predictor of cardiovascular risk factors^26,27^, our results from the development dataset showed better linear relationship between whole-body fat percentage and height-to-waist ratio (the foundation of RFM) versus waist-to-height ratio among women, men, across ethnic groups, and age categories (Supplementary Tables 5 and 6). It should be noted that for body fat estimation purposes, the useful waist-to-height ratio is not an intuitive surrogate of whole-body fat percentage.

In our validation dataset, we found a high rate of false negative cases (low sensitivity) of body fat-defined obesity when using BMI at the cut-points arbitrarily chosen, both among women and men. These findings are consistent with those from previous studies, regardless the DXA body fat cut-points used to define obesity^9,28,29^. An RFM ≥33.9 for women and ≥22.8 for men showed a high sensitivity to identify individuals with obesity, 95.0% and 96.2%, respectively. Likewise, using same cut-points, RFM had lower rates of obesity total misclassification than BMI among all women and all men and among Mexican-Americans (8.2%), European-Americans (11.3%) and African-Americans (~9.9%), indicating a consistent and relatively low rate of obesity misclassification with RFM across these ethnic groups.

The lower rates of obesity misclassification with RFM compared with BMI (among women: ~13% vs. ~57%, respectively; among men: ~9% vs. ~13%), supports the clinical utility of RFM to identify individuals with high body fat percentage, a condition that has been associated with increased mortality^1–3^. Overall, our data show that the lower rates of obesity total misclassification with RFM are largely due to the higher sensitivity (lower false negatives) of RFM for body fat-defined obesity among women and men, supporting the potential of RFM as a screening tool for obesity. Compelling evidence indicates lifestyle intervention in adult individuals with overweight or obesity may reduce morbidity and all-cause mortality^29,30^. Thus, one important aspect will be to evaluate whether early lifestyle intervention in individuals with high body fat percentage assessed by RFM could offer clinical benefits to reduce morbidity and mortality in the short and long term.

One limitation of previous studies proposing predicting equations of body fat percentage is the lack of information on the diagnostic accuracy for high body fatness^19,20,25^. In the present study, RFM showed better diagnostic accuracy for body fat-defined obesity among men compared with BMI and the CUN-BAE, Gallagher and Deurenberg equations. Among women, RFM had similar diagnostic accuracy for obesity than BMI and CUN-BAE, Gallagher, Deurenberg and Kagawa equations. Thus, one benefit of using RFM over BMI is its relatively high diagnostic accuracy for obesity in both sexes (AUC ≥ 0.93). An additional advantage of RFM over BMI was its superior diagnostic accuracy for diabetes, a well-established cardiovascular risk factor^31^. RFM also showed superior diagnostic accuracy for diabetes relative to CUN-BAE and Gallagher equations among women. Our findings are consistent with meta-analyses of numerous cross-sectional studies, concluding waist-to-height ratio is superior to BMI to identify cardiovascular risk factors, including diabetes^26,27^.

Measurement of waist circumference is unstandardized and subject to variability. However, measurement error due to the anatomical placement of measuring tape appears to have little effect on the association between waist circumference and cardiovascular risk factors, including diabetes^32^. Moreover, the reproducibility between measurements is very high^33^. Nevertheless, if waist circumference measurements become part of routine clinical evaluation, it should be implemented with adequate tools and professional training.

The present study has some limitations. (1) We used DXA as the reference method. Compared with the four-compartment method, DXA underestimates fat percentage in the lower ranges and in men, and overestimates fat percentage in the higher ranges and in women^34,35^. Thus, the performance of RFM could well be slightly superior or inferior to the actual estimates depending on the relative fat mass and sex. (2) NHANES data analysis by ethnicity was limited to Mexican-American, European-American, and African-American adult individuals. Therefore, our results cannot be extrapolated to other ethnic groups. Future studies will be required to evaluate the performance of RFM in other ethnicities (e.g. Asians and Native-American populations) as well as in children, athletes, and in individuals with specific diseases. (3) Our study was cross-sectional and used a single-point measurement of each anthropometric. Thus, our study was not designed to propose RFM cut-points for the diagnosis of obesity. We defined obesity using arbitrary cut-points of DXA-measured body fat percentage to compare obesity misclassification by RFM and BMI. Sensitivity analysis showed RFM had better diagnostic accuracy for obesity than BMI among men regardless the cut-point used to define obesity. (4) RFM validation was limited to a nationally representative sample of the US population. External validation of the RFM performance and obesity misclassification with RFM in populations from other countries are warranted.

Our findings showed RFM equation, which is based on height/waist, had superior performance (accuracy and precision) to BMI and the CUN-BAE, Gallagher, Deurenberg and Kagawa equations to estimate whole-body fat percentage in women and men. Overall, total misclassification of body fat-defined obesity with RFM was lower than with BMI among women and men, across ethnic groups, including Mexican-Americans, European-Americans and African-Americans. We conclude that, in the population studied, RFM was more accurate than BMI to estimate whole-body fat percentage among women and men and improved body fat-defined obesity misclassification among American adult individuals of Mexican, European or African ethnicity.

## Methods

### Study population

NHANES is a program designed to study the health and nutritional status of the non-institutionalized population of the United States. NHANES is conducted annually and released in two-year cycles using a nationally representative sample across the country, selected using a multistage, probability sampling design. NHANES 1999–2004 and NHANES 2005–2006 oversampled Mexican-American and African-American populations to obtain representative samples of these ethnic groups for reliable estimates^36^. Thus, analysis by ethnic groups were limited to Mexican-American, European-American (White) and African-American individuals.

The present study did not require approval or exemption from the Cedars-Sinai Medical Center Institutional Review Board as it involved the analysis of publicly available de-identified data only.

### Data

An advantage of using NHANES for the present study is that it constitutes the largest database containing information on whole-body composition for the US population, which was collected between 1999 and 2006 using the well accepted method DXA^37,38^. Thus, DXA was used as the reference method to measure whole-body fat percentage.

NHANES 1999–2004 was used as the development dataset. Multiple imputation was applied to replace missing DXA data^39^. Details are provided in the Supplementary Material. Model development included individuals 20 to 85 years of age. In total, 12,581 observations were included for model development (Fig. 1).

NHANES 2005–2006 was used as the validation dataset. Multiple imputation was also used to account for missing data (see Supplementary Material). Model validation included individuals 20 to 69 years of age, as DXA was performed only on individuals 69 years old and younger in this sample. In total, 3,456 observations were included for model validation (Fig. 1).

### Anthropometric measurements

Waist circumference was measured placing the measuring tape around the trunk (unclothed waist) in a horizontal plane at the level of the uppermost lateral border of the right ilium during standing position at the end of the expiration. The measurement was recorded to the nearest 0.1 cm. Body weight was measured with an electronic scale (examinee wearing underwear only). Height was measured with an electronic stadiometer^40^. Other anthropometrics were measured using standard procedures^40^.

### DXA scans

DXA scans were acquired using a Hologic QDR 4500A fan-beam densitometer (Hologic, Inc., Bedford, Massachusetts) and Hologic DOS software version 8.26:a3*. Scans were reviewed and analyzed by the University of California, San Francisco, using Hologic Discovery software, version 12.1 for NHANES 1999–2004 and version 12.4 for NHANES 2005–2006^39^. Body fat percentage was calculated as the ratio of DXA whole-body fat mass (g) to DXA whole-body total mass (g), multiplied by 100.

### Model development and selection

Common anthropometrics including body weight, height, triceps and subscapular skinfolds, arm and leg lengths, and waist, calf, arm and thigh circumferences were tested for correlation with DXA-measured whole-body fat percentage in men and women, independently. Simple and combined anthropometrics that had the highest correlation with body fat percentage among women and men, independently, were the foundation for our model development using linear regression for survey data. We also explored the effect of adding age and ethnicity in the regression models. Two- and three-way anthropometric indices were generated, including combination of integer powers, square root, and reciprocal transformations. Model selection was based on the ability to predict whole-body fat percentage (R^2^) in both women and men and sex-ethnicity subgroups, the lowest RMSE, the lowest Akaike information criterion^41^, the overall performance in terms of accuracy and precision, and the simplicity to estimate body fat percentage in both women and men. Further details are provided in the Supplementary Material.

### Model validation

Validation of the final model was performed in NHANES 2005–2006. RFM performance was validated in the participants of the NHANES 2005–2006, a large nationally representative sample of the US adult population but different sample from the development dataset. Development and validation datasets were combined into one dataset (NHANES 1999–2006, n = 16,037 adult individuals) to perform internal validation using the most accepted technique, the bootstrapping, to obtain bootstrapped standard errors and verify the statistical differences between selected models and BMI^42^.

### Model performance

We used concordance correlation coefficient and Bland-Altman plots to examine the agreement between estimated and DXA-measured body fat percentage^43^. Bias was calculated as the median difference between estimated and measured body fat percentage. For the purpose of the present study, accuracy (how closely an individual estimate agrees with the “true” or reference value) was calculated as the proportion of cases with <20% difference between estimated and DXA-measured whole-body fat percentage^44^. Precision was calculated as the interquartile range of the difference between estimated and measured body fat percentage^44^. The performance of our final model was compared with four published equations that are based on age and BMI or waist-to-height ratio reported to have a high prediction for body fat percentage: Gallagher^25^, CUN-BAE^20^, Deurenberg^45^ and Kagawa equations^46^.

### Obesity misclassification

To date, there is no consensus on the diagnosis of obesity based on body fat percentage. Thus, to define obesity based on body fat percentage we used arbitrary cut-points of DXA-measured body fat percentage: ≥33.9% for women and ≥22.8% for men (corresponding cut-points between the first and second quintiles for each sex). Misclassification of body fat-defined obesity was expressed as false negative rate (1–sensitivity), false positive rate (1–specificity), and total misclassification error (the proportion of false positives and false negatives together among all women, all men, and among both sexes combined).

### Diagnostic accuracy for obesity and diabetes

Diabetes was defined if an individual had a measured glycated hemoglobin ≥6.5% or a fasting plasma glucose ≥126 mg/dL or self-reported diagnosed diabetes^47^. Diagnostic accuracy for obesity and diabetes were estimated using the receiver-operating-characteristic curve analysis, expressed as the AUC^48^.

### Statistical analysis

We used clusters and strata information and probability weights for all analyses to account for the complex design of the NHANES^49^. Estimates of the Akaike information criterion and concordance correlation coefficient were adjusted for probability weights only. Initial examination of the association between body fat percentage and anthropometrics, including those generated in the present study, were performed using unweighted data. Listwise deletion was used to handle missing data for correlation analyses. Pooled data estimates (and their 95% confidence intervals) were obtained using Rubin’s equations^50^ implemented in STATA for analysis of multiple imputation in complex survey data. Variance estimates for development and validation datasets were obtained using Taylor series linearization. Bootstrapping with 1000 replicates was used to obtain confidence intervals for adjusted R-squared and RMSE in the development and validation datasets and to perform internal validation. Wald test was used to test for interaction of ethnicity and age category with selected indices on the prediction of body fat and to calculate P values to evaluate the accuracy and diagnostic accuracy (AUC) between models^51^. Bonferroni correction was applied for multiple comparisons. All analyses were performed using Stata 14 for Windows (StataCorp LP, College Station, TX). P values were set to a two-tailed alpha level of 0.05.

## Electronic supplementary material

Supplementary Material
