# Supplementary material for: Relative fat mass (RFM) as a new estimator of whole-body fat percentage ─ A cross-sectional study in American adult individuals
Source: Sci Rep. 2018 Jul 20;8:10980. doi: 10.1038/s41598-018-29362-1 (PMC6054651; doi:10.1038/s41598-018-29362-1)
Supplement: Supplementary file 1 — Supplementary Material [file 41598_2018_29362_MOESM1_ESM.pdf]

Supplementary Material

Relative fat mass (RFM) as a new estimator of whole-body fat percentage ─ A cross-sectional study in American adult individuals

Orison O. Woolcott, MD*, Richard N. Bergman, PhD

Sports Spectacular Diabetes and Obesity Wellness and Research Center, Cedars-Sinai Medical Center,

Los Angeles, CA 90048, USA

Running title: Estimation of body fat percentage

*Corresponding author:

Orison O. Woolcott, MD
Sports Spectacular Diabetes and Obesity Wellness and Research Center
Cedars-Sinai Medical Center
8700 Beverly Boulevard, Thalians E103
Los Angeles, CA 90048
United States of America
Telephone: +1 (310) 423-2926
Fax: +1 (310) 967-3869
Email: Orison.Woolcott@cshs.org

Table of Contents

Supplementary Methods 3

Supplementary Figure 1. Prediction of whole-body fat percentage using anthropometric-derived equations among participants in NHANES 1999-2004 (development dataset). 4

Supplementary Figure 2. Variability of anthropometrics and body composition by age among participants of the NHANES 1999-2004. 5

Supplementary Figure 3. Bland-Altman plots showing the agreement between whole-body fat percentage estimated by the height/waist equation (Relative Fat Mass, RFM) and that measured by DXA among participants of the NHANES 1999-2004 (development dataset). 6

Supplementary Figure 4. Performance of height/waist equation (RFM) by sex and ethnicity in NHANES 2005-2006 (validation dataset). 7

Supplementary Figure 5. Performance of height/waist equation (RFM) by age category in NHANES 2005-2006. 8

Supplementary Figure 6. Performance of height/waist equation (RFM) by quintiles of body fat percentage in NHANES 2005-2006. 9

Supplementary Figure 7. Performance of height/waist equation (RFM) among women and men in NHANES 1999-2006, by age category. 10

Supplementary Figure 8. Performance of height/waist equation (RFM) among women and men in NHANES 1999-2006, across body fat categories. 11

Supplementary Figure 9. Diagnostic accuracy for high whole-body fat percentage in NHANES 1999-2006. 12

Supplementary Figure 10. Receiver operating characteristic curves for diabetes among participants of the NHANES 1999-2006. 13

Supplementary Table 1. Frequency of DXA multiply imputed data in among adult participants (≥20 years old) in the development dataset (NHANES 1999-2004). 14

Supplementary Table 2. Frequency of DXA multiply imputed data among adult participants (20-69 years old) in the validation dataset (NHANES 2005-2006). 15

Supplementary Table 3. Correlation matrix (unweighted Pearson's r) between DXA-estimated whole-body fat percentage and common anthropometrics among adult individuals (≥20 years old) in the development dataset. 16

Supplementary Table 4. Correlation matrix (unweighted Pearson's r) between DXA-estimated whole-body fat percentage and more than 350 indices derived using common anthropometrics among adult individuals (≥20 years old) in the development dataset. 17

Supplementary Table 5. Prediction of DXA-estimated whole-body fat percentage by sex and ethnicity among adult participants in NHANES 1999-2004.* 29

Supplementary Table 6. Prediction of whole-body fat percentage in NHANES 1999-2004, by age category*. 31

Supplementary Table 7. Raw equations developed for the prediction of DXA-estimated whole-body fat percentage among participants (≥20 years old) in NHANES 1999-2004. 32

Supplementary Table 8. Concordance correlation coefficients (rho_c) between predicted and DXA-estimated body fat percentage by sex and ethnicity in NHANES 1999-2004.* 33

Supplementary Table 9. Performance of selected regression model equations for the estimation of body fat percentage in NHANES 1999-2004.*.......... 34

Supplementary Table 10. Prediction of body fat percentage in NHANES 2005-2006, by sex and ethnicity*. 36

Supplementary Table 11. Prediction of body fat percentage in NHANES 1999-2006, by sex and ethnicity*. 38

Supplementary Table 12. Prediction of body fat percentage in NHANES 1999-2006, by age category*. . 40

Supplementary Table 13. Performance of BMI and RFM in NHANES 1999-2006.......... 41

Supplementary Table 14. Performance of BMI, RFM and published equations for the estimation of whole-body fat percentage across age categories in NHANES 1999-2006.......... 42

Supplementary Table 15. Model fitting of BMI and RFM for body fat percentage, fat mass and trunk fat percentage in NHANES 1999-2006*.......... 43

Supplementary Table 16. Comparison of diagnostic accuracy for obesity among BMI, RFM and previously published equations among participants of NHANES 1999-2006. 44

References.......... 45

Supplementary Methods

Development dataset (NHANES 1999-2004): A total of 31,126 individuals of all ages were surveyed. Individuals who were 85 years and older were codified as having and age of 85 years. Model development included individuals 20 to 85 years of age, who were eligible participants for Dual Energy X-ray Absorptiometry (DXA) scans. Females 59 years old and younger who had positive pregnancy test at the time of the examination or those with self-reported pregnancy, even with negative pregnancy test, were excluded. Individuals who received radiographic contrast material in the past 7 days or had a self-reported weight over 300 pounds or height over 6’5’’ were also excluded. Scans that could not be analyzed accurately were set to missing. Since missing DXA values were not considered to occur at random, NHANES used random multiple imputation to replace missing data with imputed values (five multiply imputed data were generated per observation). Multiply imputed values with extreme variability were set to missing and excluded. Observations with missing body weight, height or waist circumference were excluded from the dataset. However, to maximize the power of our analyses we did not exclude observations with missing data on triceps and subscapular skinfolds, arm and leg lengths, or calf, arm and thigh circumferences. Individuals were stratified into age categories according to the recommendations for NHANES 1999–2004 sample design [1].

Validation dataset (NHANES 2005-2006): A total of 10,348 individuals of all ages were surveyed. Eligible participants for DXA scans were individuals 20 to 69 years of age. Same exclusion criteria and imputation method were applied as for NHANES 1999-2004.

Models development, selection, and performance: In the development dataset, as a first analysis, simple anthropometrics including body weight, height, triceps and subscapular skinfolds, arm and leg lengths, and waist, calf, arm and thigh circumferences were tested for correlation with DXA-measured whole-body fat percentage in men and women, independently. Separate analyses in women and men were performed based on a known gender difference in body fat percentage [2,3]. The goal of the present study was to identify the simplest equation that is more accurate than the BMI to estimate whole-body fat percentage. Thus, we were focused in generating models based on no more than three anthropometrics using linear regression. Two- and three-way anthropometric indices were empirically generated, including a combination of integer powers (taken to the power 1, 2 and 3, and in some cases to the power 4 if we noted improved correlation with measured body fat percentage), square root, and reciprocal, but not logarithmic, transformations. Multiple linear regression was also used to further identify possible additional combinations of anthropometrics, and explore the effect of age and ethnicity as individual variables or interaction terms with anthropometrics in the models, to achieve the highest prediction of body fat percentage in women and men, independently and together. An increase in adjusted R-squared by 0.02 was considered meaningful to consider the inclusion of age or ethnicity in the models.

Coefficients in the final model equations were rounded to integers for practical purposes if concordance correlation coefficient (rho_c, a measure of agreement) was not substantially affected (a decrease in rounded rho_c less than 0.01). In addition, we conducted a preliminary screening on rounded equations using imputation 1 and compared with the performance of raw equations. Rounding of model coefficients was manually performed to obtain the simplest and most practical linear equation without compromising their predicting ability or performance (accuracy and precision) compared with those from their raw version.

Supplementary Figure 1. Prediction of whole-body fat percentage using anthropometric-derived equations among participants in NHANES 1999-2004 (development dataset).

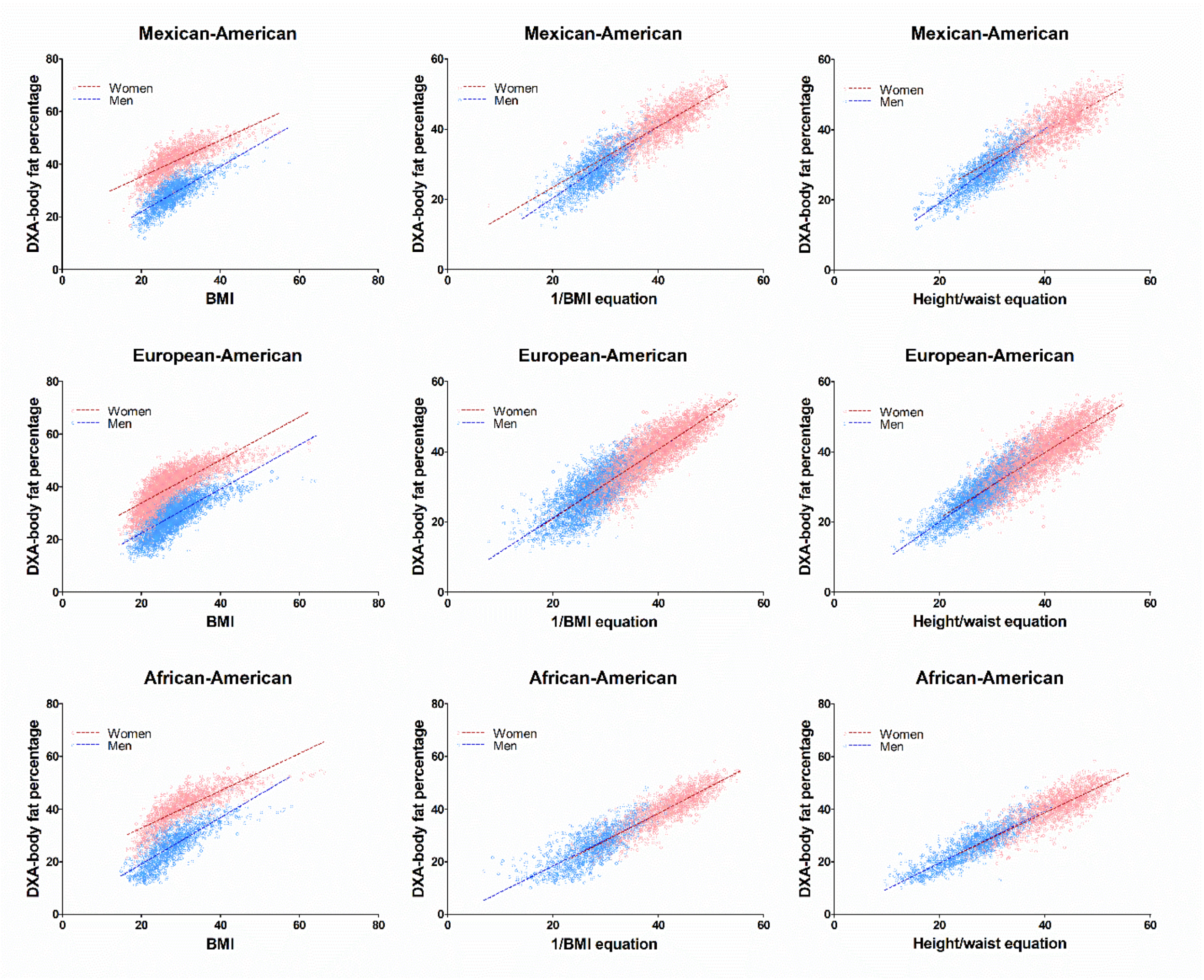

Among simplest models selected, height/waist equation showed higher adjusted R^2 and lowest RMSE than BMI among Mexican-American, European-American and African-American men (see Table S5 for details). Data plots correspond to DXA imputation 1.

Supplementary Figure 2. Variability of anthropometrics and body composition by age among participants of the NHANES 1999-2004.

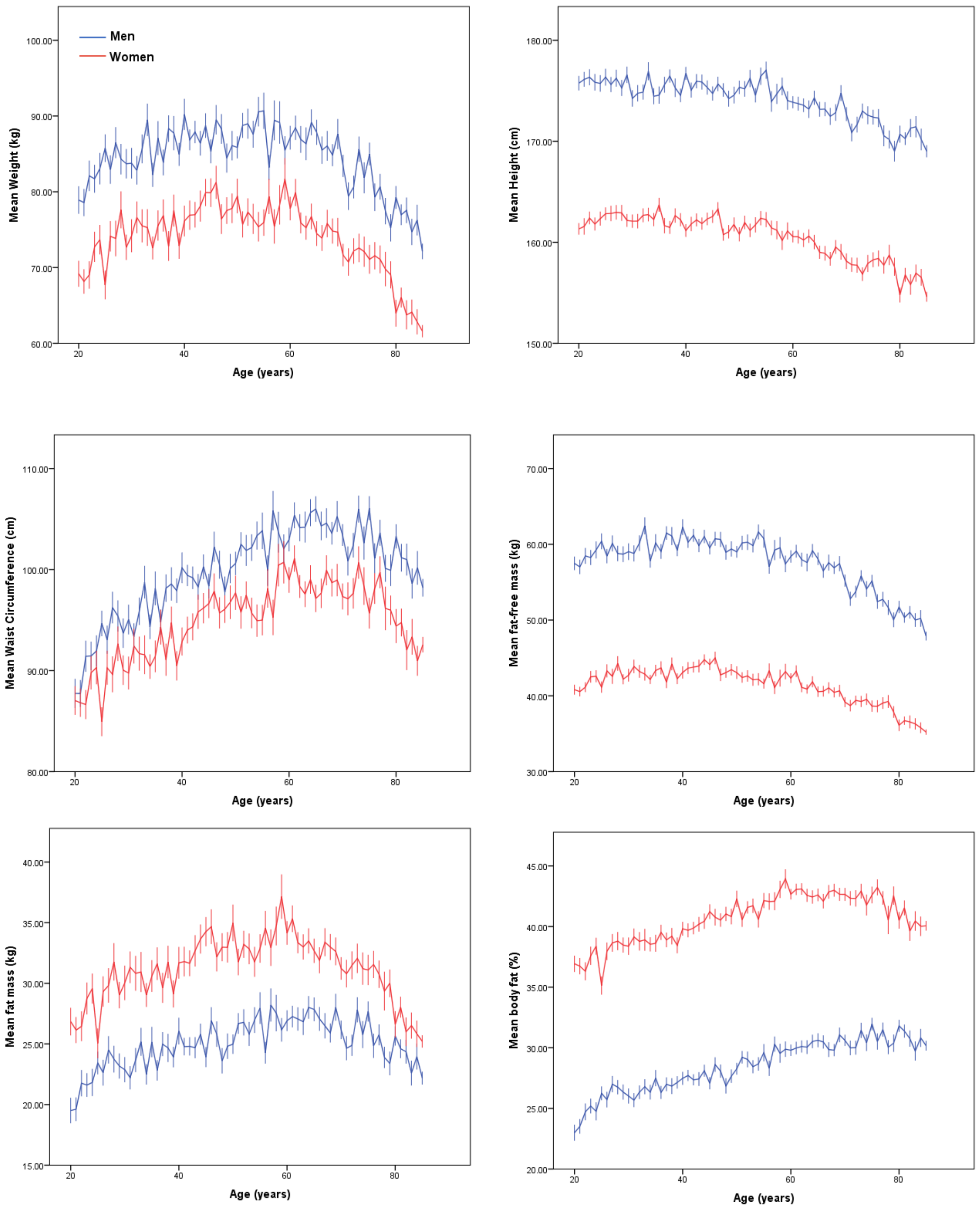

Data plots of body composition correspond to DXA imputation 1. Error bars are standard error.

Supplementary Figure 3. Bland-Altman plots showing the agreement between whole-body fat percentage estimated by the height/waist equation (Relative Fat Mass, RFM) and that measured by DXA among participants of the NHANES 1999-2004 (development dataset).

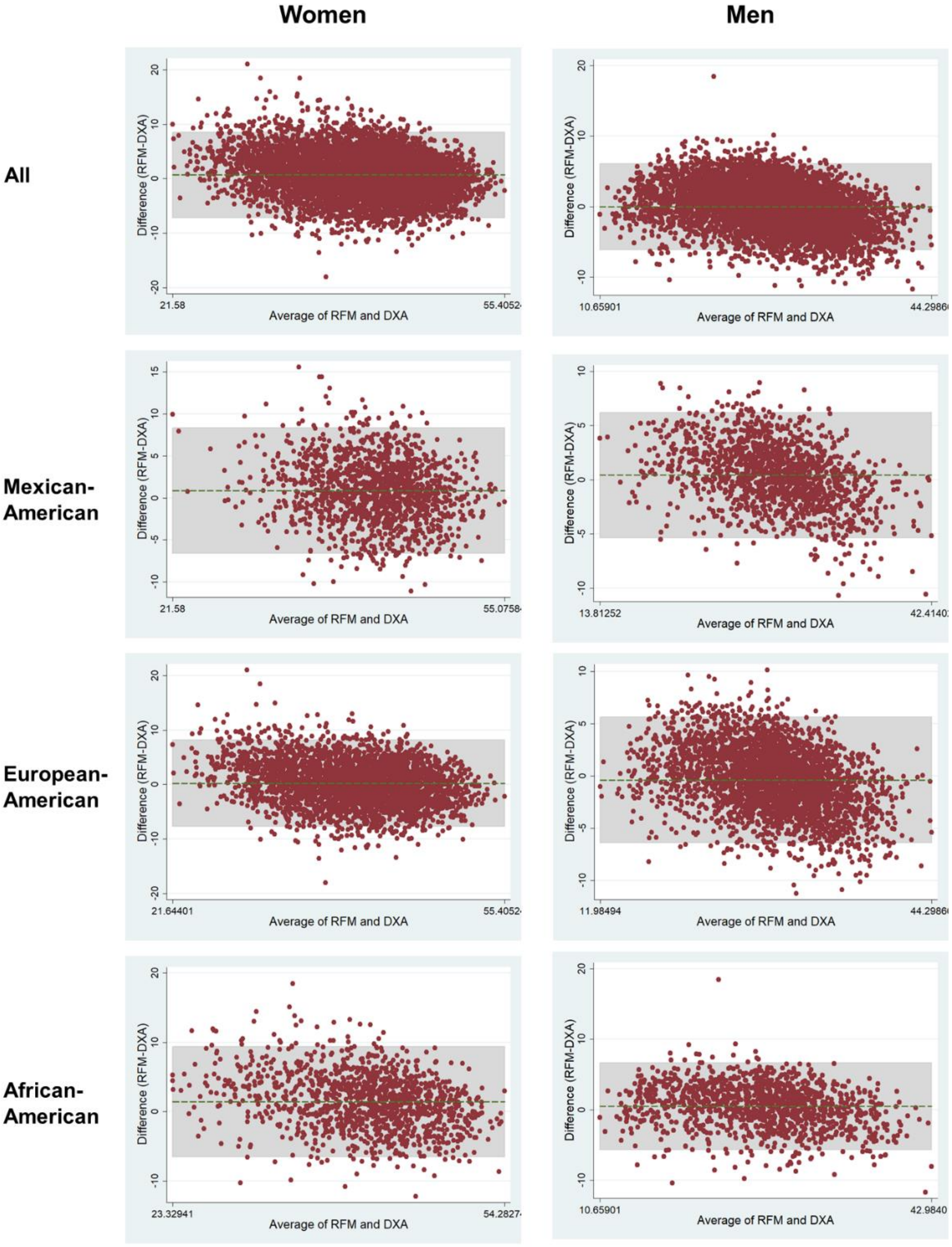

Gray bands indicate 95% limits of agreement. Green dotted lines represent the mean. Data plots correspond to DXA imputation 1.

Supplementary Figure 4. Performance of height/waist equation (RFM) by sex and ethnicity in NHANES 2005-2006 (validation dataset).

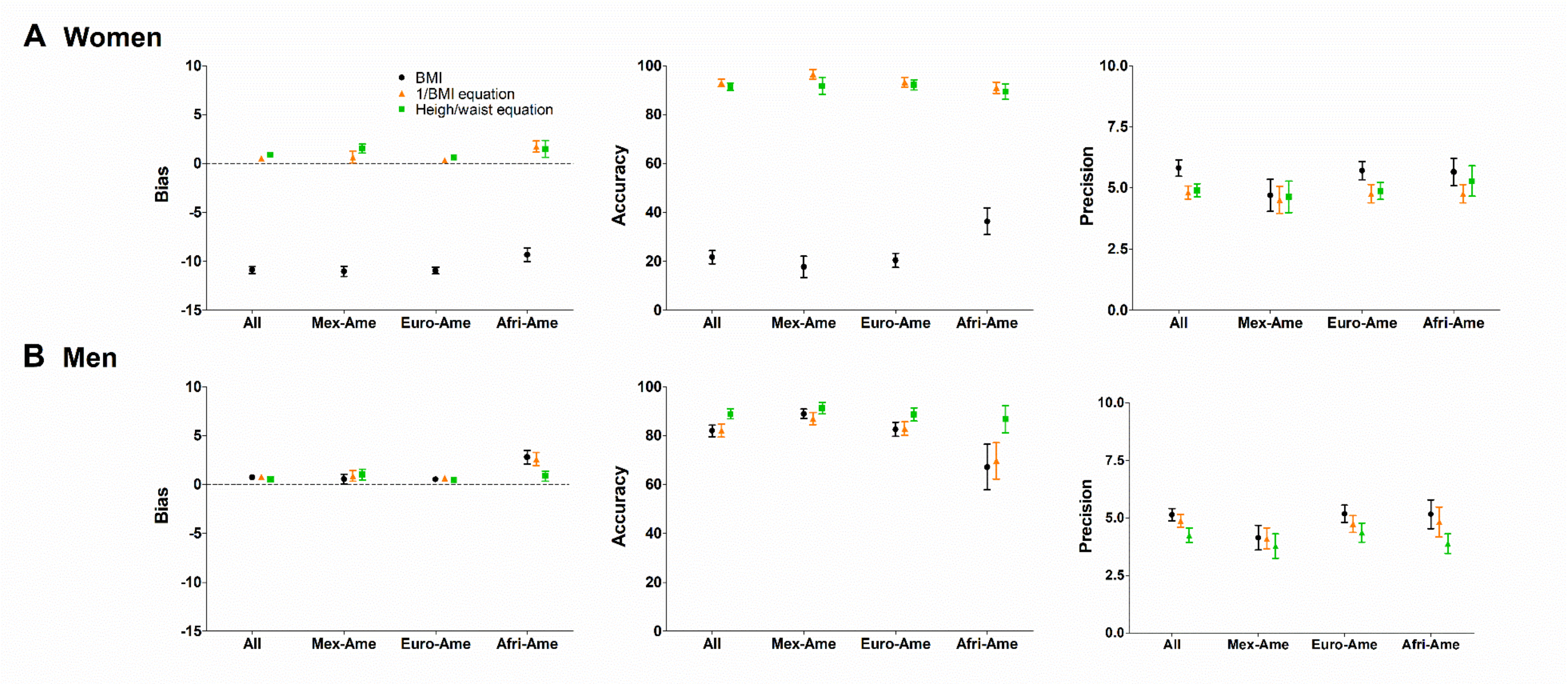

Among women, height/waist equation (relative fat mass, RFM) showed lower bias, higher accuracy and better precision than BMI Among men, RFM also showed higher accuracy and better precision than BMI. Overall, RFM showed better performance than BMI across ethnicities.

Supplementary Figure 5. Performance of height/waist equation (RFM) by age category in NHANES 2005-2006.

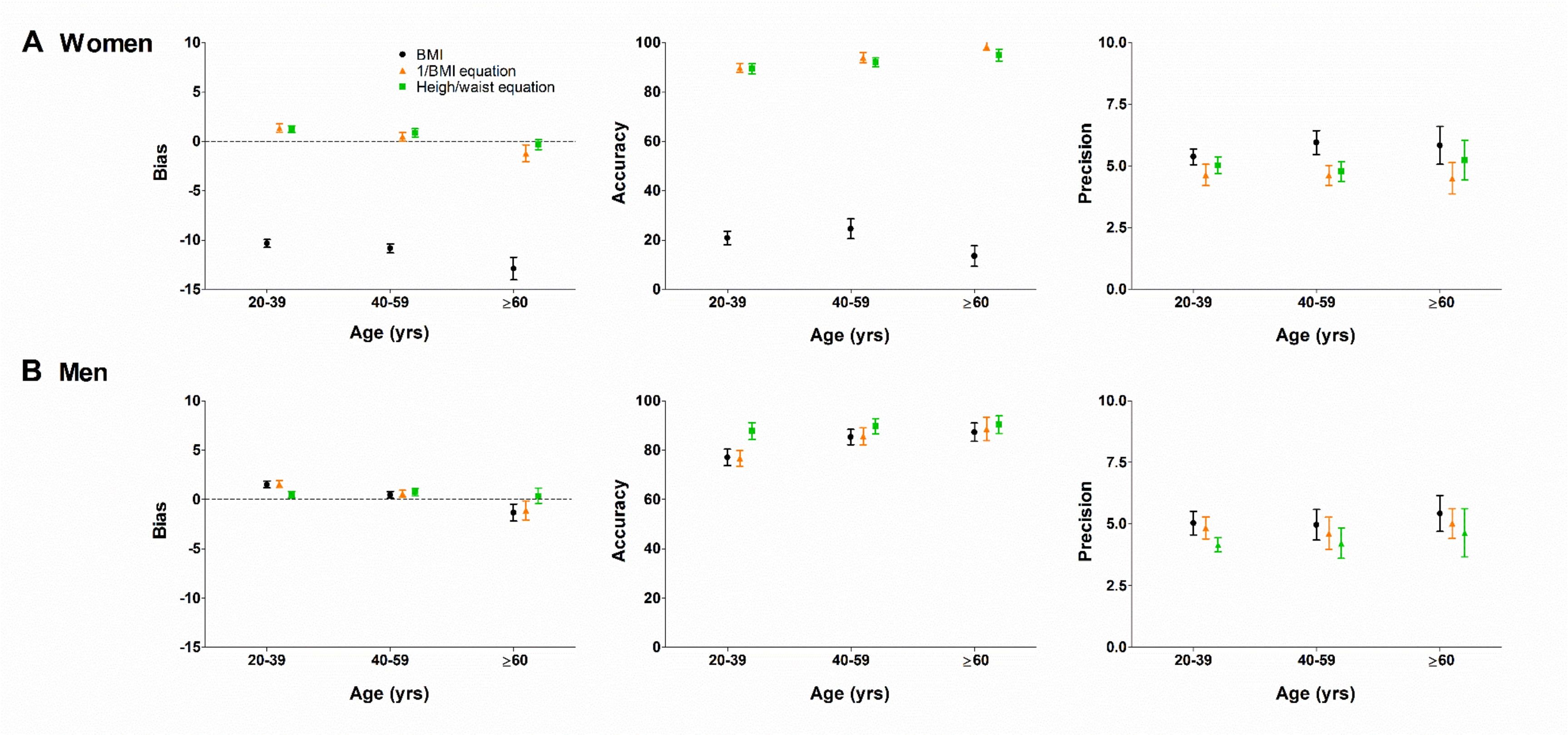

The accuracy of BMI to estimate body fat percentage among women was poor across age categories. RFM had an accuracy above 85% among young, middle-age and elderly individuals. Although 1/BMI equation showed similar accuracy than RFM among women, the accuracy of 1/BMI equation among men was lower. RFM was more precise than BMI among middle-aged women.

Supplementary Figure 6. Performance of height/waist equation (RFM) by quintiles of body fat percentage in NHANES 2005-2006.

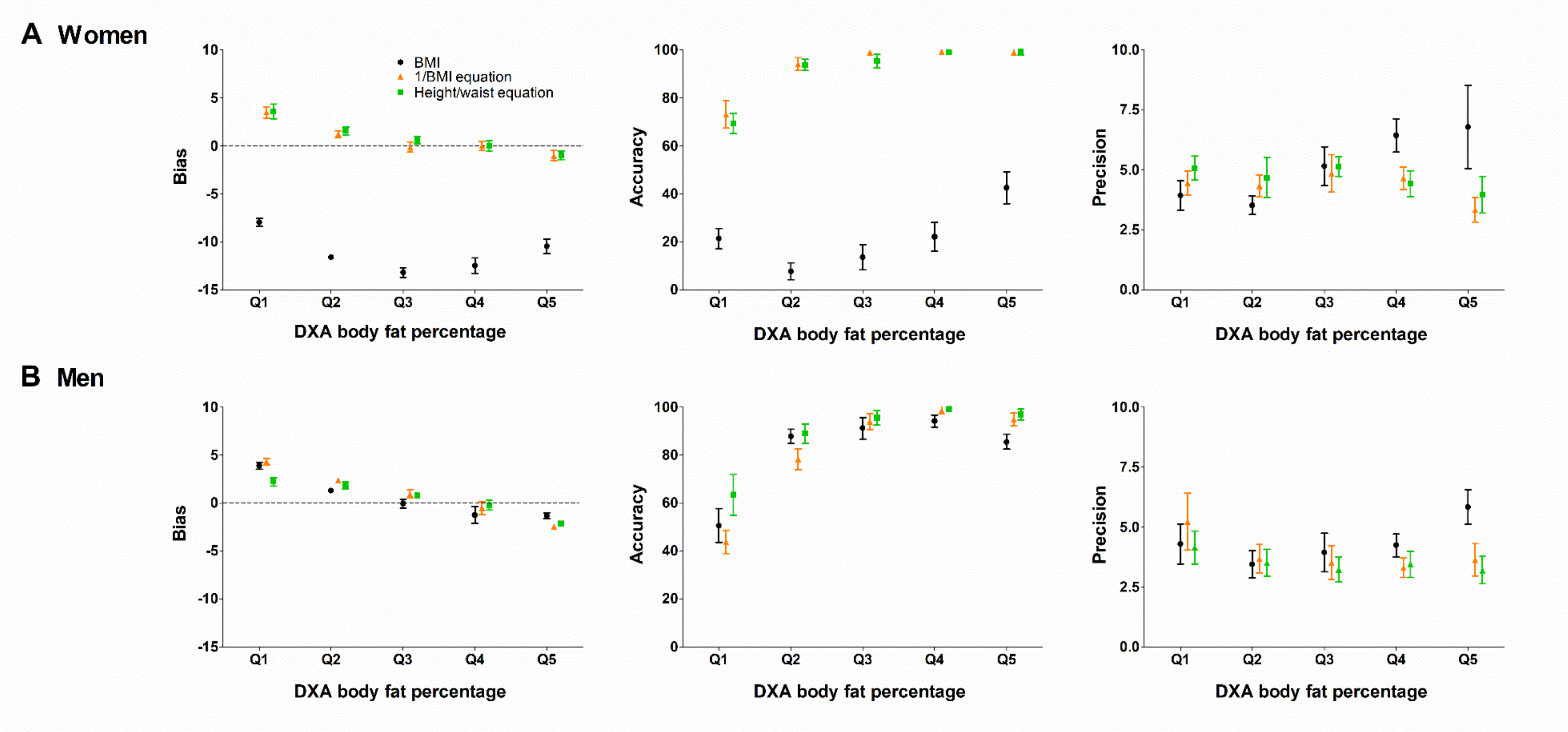

Accuracy of RFM to estimate whole-body fat percentage was overall better than BMI across body fatness among women and men. However, accuracy was lower among lean individuals. Q, quintile. For women: Q1: <33.9%; Q2: ≥33.9% to 38.4%; Q3: ≥38.5% to 42.0%; Q4: ≥42.1% to 45.6%; Q5: ≥45.7%. For men: Q1: <22.8%; Q2: ≥22.8% to 26.5%; Q3: ≥26.6% to 29.4%; Q4: ≥29.5% to 33.0%; Q5: ≥33.1%.

Supplementary Figure 7. Performance of height/waist equation (RFM) among women and men in NHANES 1999-2006, by age category.

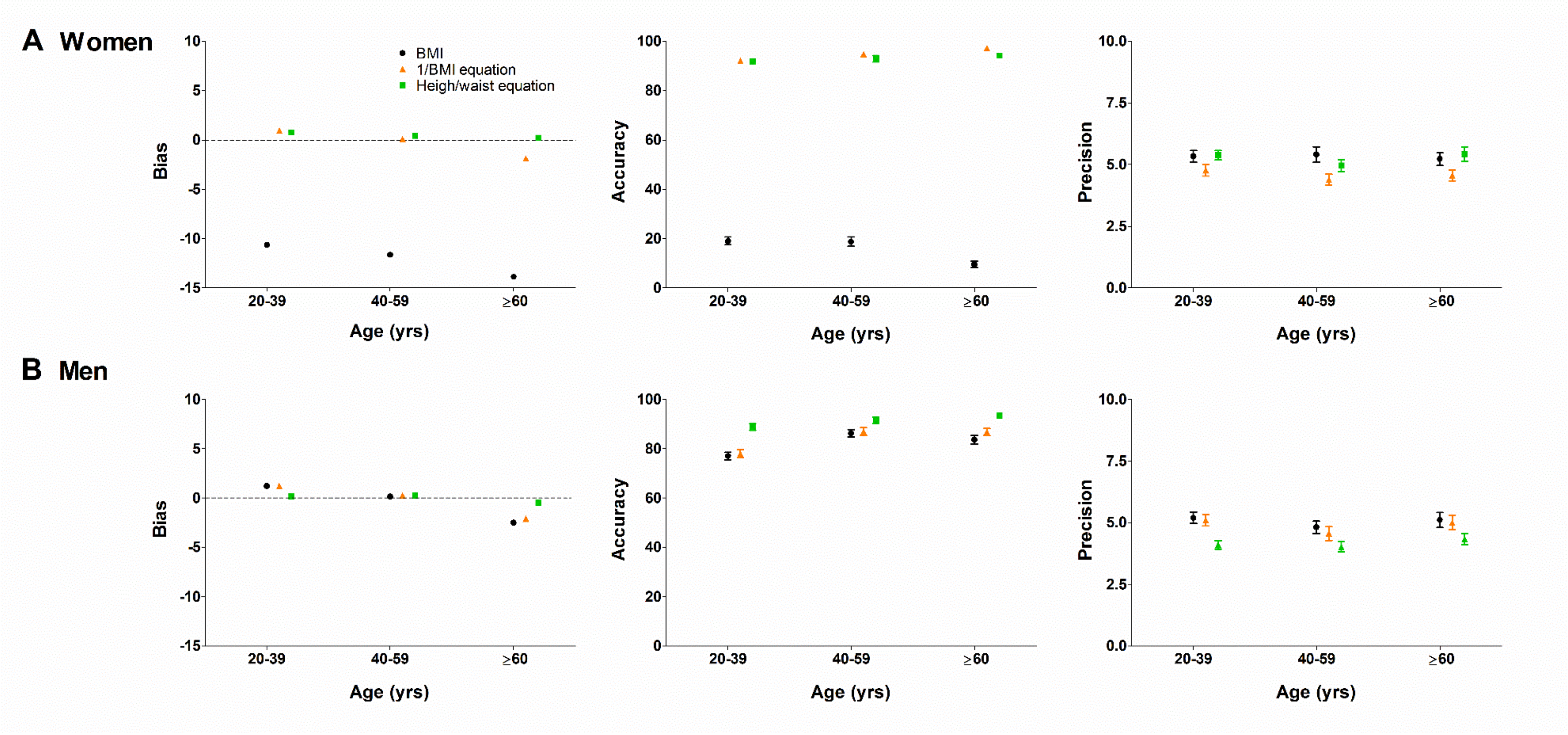

Accuracy of RFM was ~90%, superior to BMI among young, middle-age and elderly individuals, also higher than 1/BMI equation among men. RFM was also more precise than BMI and 1/BMI equation among men.

Supplementary Figure 8. Performance of height/waist equation (RFM) among women and men in NHANES 1999-2006, across body fat categories.

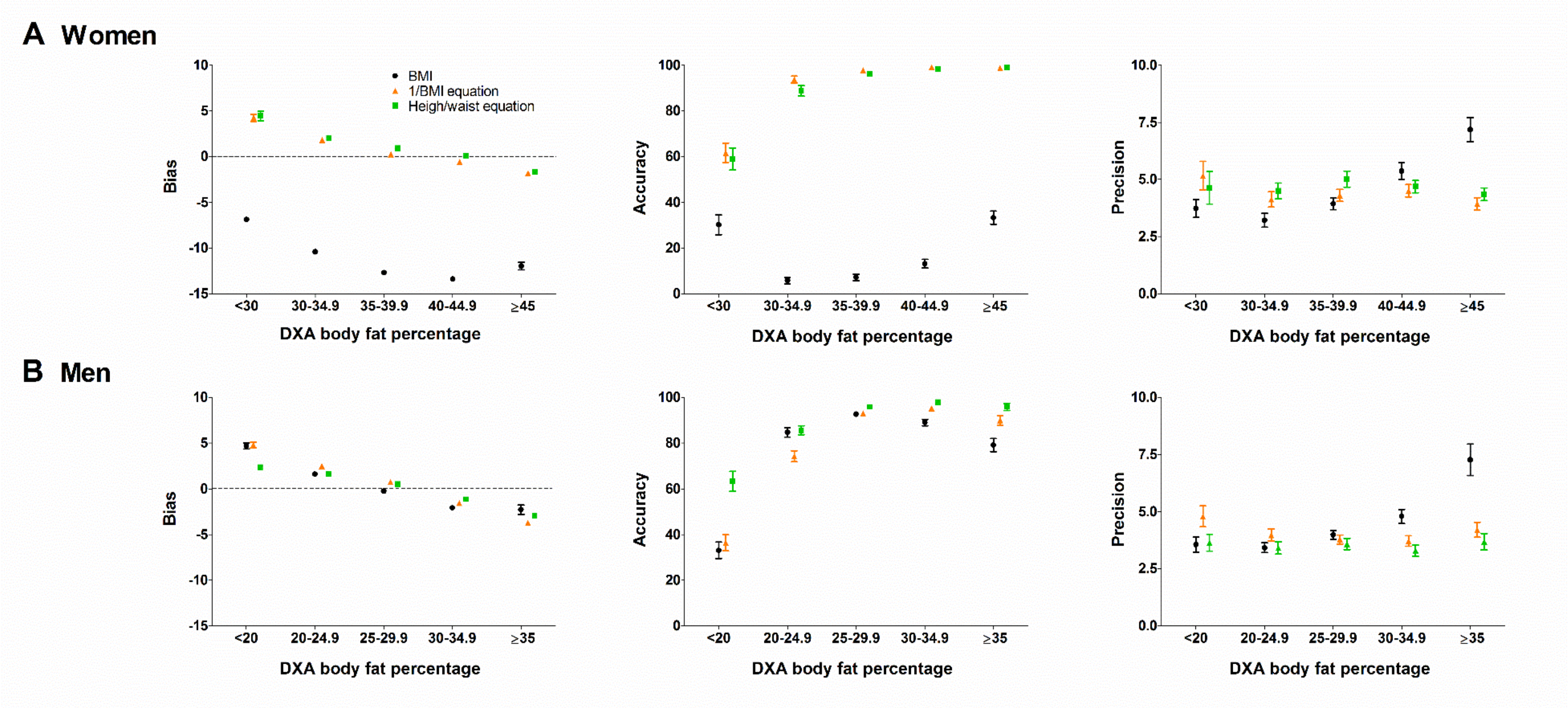

The accuracy of RFM was consistently higher than BMI across all ranges of body fatness among women and men. However, the accuracy of RFM was below 65% among women with a body percentage below 30% and among men with a body fat percentage below 20%.

Supplementary Figure 9. Diagnostic accuracy for high whole-body fat percentage in NHANES 1999-2006.

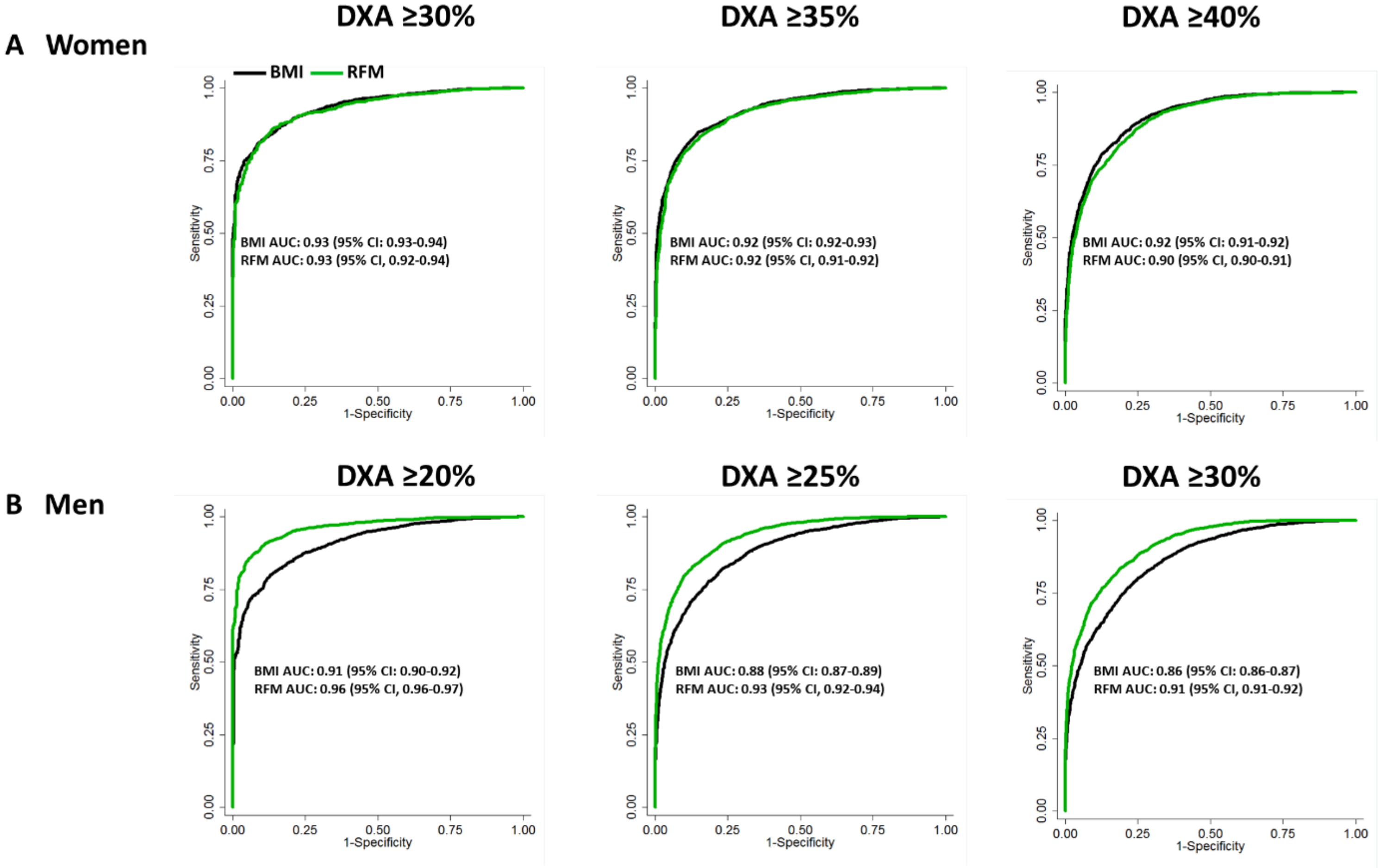

Plots show comparison of diagnostic accuracy for body fat excess using different cut-points of DXA-measured body fat percentage. Among women, RFM showed similar accuracy (expressed as the area under the curve (AUC)) to BMI to identify individuals with a body fat percentage at or above 30% (P=0.19). BMI showed marginal better accuracy to RFM to identify individuals with a body fat percentage at or above 35% (P=0.001) and at or above 40% (P<0.001). Among men, RFM showed clearly higher accuracy than BMI to identify individuals with a body fat percentage at cut-off values of 20% (P<0.001), 25% (P<0.001), and 30% (P<0.001). Values represent pool weighted estimates. Data plots correspond to DXA imputation 1.

Supplementary Figure 10. Receiver operating characteristic curves for diabetes among participants of the NHANES 1999-2006.

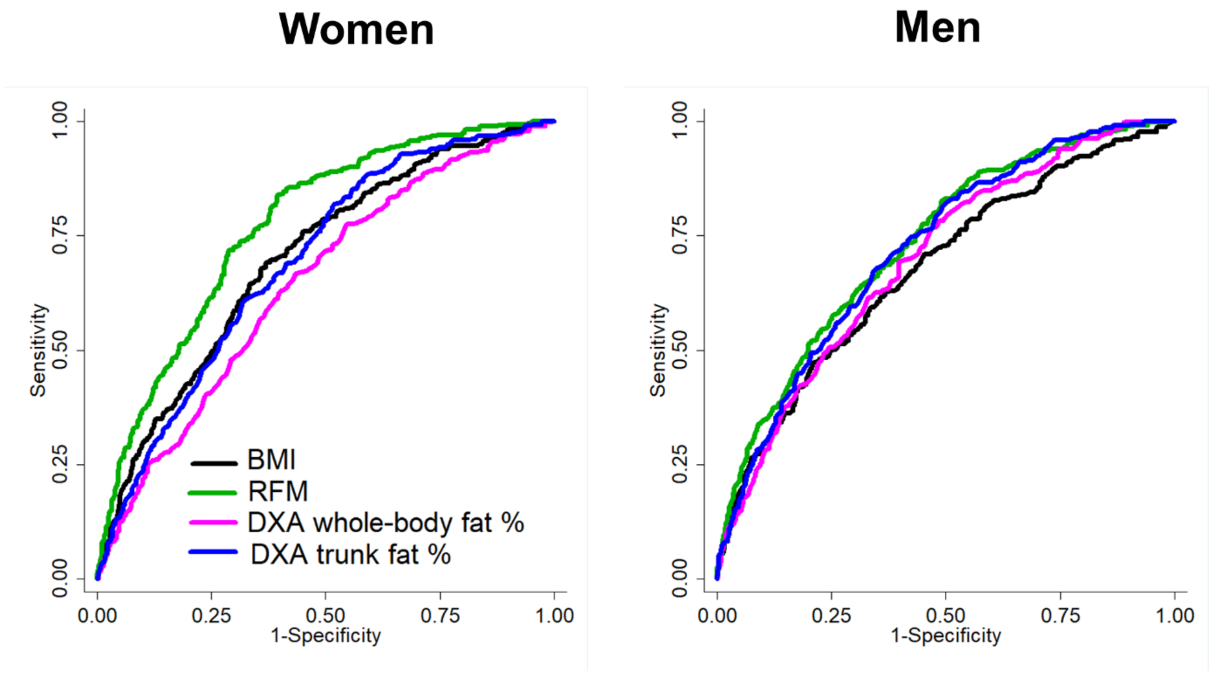

Plots show comparison of diagnostic accuracy (expressed as the AUC) among RFM, BMI, DXA-measured whole-body fat percentage and DXA-measured trunk fat percentage for diabetes. Among women, RFM showed superior accuracy than DXA-measured trunk fat percentage and whole-body fat percentage to identify diabetes cases (P<0.001 for both comparisons). Among men, RFM showed superior diagnostic accuracy than whole-body fat percentage (P=0.001) but not than trunk fat percentage (P=0.548). All P values were Bonferroni-adjusted.

Supplementary Table 1. Frequency of DXA multiply imputed data in among adult participants (≥20 years old) in the development dataset (NHANES 1999-2004).

	Non-imputed data (%)	Imputed data (%)	Total	Non-imputed data (%)	Imputed data (%)	Total
	**Women**			**Men**		
	N=4,829 (77.1)	N=1,432 (22.9)	N=6,261	N=5,065 (80.1)	N=1,255 (19.9)	N=6,320
**Ethnicity**						
Mexican-American	1,122 (80.2)	277 (19.8)	1,399	1,208 (83.9)	231 (16.1)	1,439
European-American	2,432 (78.3)	675 (21.7)	3,107	2,527 (79.1)	667 (20.9)	3,194
African-American	865 (69.5)	379 (30.5)	1,244	922 (76.7)	280 (23.3)	1,202
**Age category**						
20-39 Years old	1,600 (80.4)	389 (19.6)	1,989	1,835 (86.2)	295 (13.8)	2,130
40-59 Years old	1,580 (79.1)	418 (20.9)	1,998	1,595 (80.7)	381 (19.3)	1,976
60-69 Years old	1,649 (72.5)	625 (27.5)	2,274	1,635 (73.8)	579 (26.2)	2,214
**BMI* category**						
<18.5	102 (84.3)	19 (15.7)	121	67 (80.7)	16 (19.3)	83
18.5-24.9	1,643 (83.1)	333 (16.9)	1,976	1,606 (85.2)	280 (14.8)	1,886
25-29.9	1,567 (81.8)	349 (18.2)	1,916	2,244 (85.5)	380 (14.5)	2,624
≥30	1,517 (67.5)	731 (32.5)	2,248	1,148 (66.5)	579 (33.5)	1,727

* BMI, body mass index (weight in kilograms divided by squared height in meters).

Supplementary Table 2. Frequency of DXA multiply imputed data among adult participants (20-69 years old) in the validation dataset (NHANES 2005-2006).

	Non-imputed data (%)	Imputed data (%)	Total	Non-imputed data (%)	Imputed data (%)	Total
	**Women**			**Men**		
	N=1,280 (75.3)	N=420 (24.7)	N=1,700	N=1,346 (76.7)	N=410 (23.3)	N=1,756
**Ethnicity**						
Mexican-American	270 (76.1)	85 (23.9)	355	336 (85.1)	59 (14.9)	395
European-American	592 (77.6)	171 (22.4)	763	621 (76.6)	190 (23.4)	811
African-American	312 (71.7)	123 (28.3)	435	293 (69.3)	130 (30.7)	423
**Age category**						
20-39 Years old	517 (75.4)	169 (24.6)	686	588 (77.0)	176 (23.0)	764
40-59 Years old	534 (75.9)	170 (24.1)	704	523 (77.0)	156 (23.0)	679
60-69 Years old	229 (73.9)	81 (26.1)	310	235 (75.1)	78 (24.9)	313
**BMI category**						
<18.5	30 (85.7)	5 (14.3)	35	17 (73.9)	6 (26.1)	23
18.5-24.9	426 (80.4)	104 (19.6)	530	366 (82.8)	76 (17.2)	442
25-29.9	366 (81.7)	82 (18.3)	448	588 (83.2)	119 (16.8)	707
≥30	458 (66.7)	229 (33.3)	687	375 (64.2)	209 (35.8)	584

Supplementary Table 3. Correlation matrix (unweighted Pearson's r) between DXA-estimated whole-body fat percentage and common anthropometrics among adult individuals (≥20 years old) in the development dataset.

	BFP*	BMI	WAIST	ARMC	BW	TRI	THIGH	SUB	CALF	ARML	Height
**Women**											
Body mass index (BMI), kg/m^2	0.78										
Waist circumference (WAIST), cm	0.76	0.90									
Arm circumference (ARMC), cm	0.73	0.92	0.83								
Body weight (BW), kg	0.70	0.93	0.88	0.90							
Triceps skinfold (TRI), mm	0.65	0.67	0.58	0.74	0.66						
Thigh circumference (THIGH), cm	0.62	0.84	0.67	0.82	0.88	0.65					
Subscapular skinfold (SUB), mm	0.59	0.69	0.63	0.69	0.65	0.70	0.56				
Calf circumference (CALF), cm	0.57	0.80	0.65	0.78	0.85	0.58	0.87	0.50			
Arm length (ARML), cm	0.25	0.35	0.41	0.45	0.55	0.26	0.43	0.23	0.44		
Height, m	−0.11	−0.02†	−0.07	0.11	0.32	0.07	0.24	0.00†	0.28	0.61	
Leg length, cm	−0.11	−0.05	−0.06	0.04‡	0.18	0.09	0.30	0.02†	0.24	0.44	0.63
**Men**											
Body mass index (BMI), kg/m^2	0.74										
Waist circumference (WAIST), cm	0.83	0.91									
Arm circumference (ARMC), cm	0.53	0.87	0.74								
Body weight (BW), kg	0.63	0.91	0.88	0.88							
Triceps skinfold (TRI), mm	0.71	0.69	0.68	0.62	0.65						
Thigh circumference (THIGH), cm	0.45	0.81	0.65	0.86	0.86	0.57					
Subscapular skinfold (SUB), mm	0.62	0.69	0.67	0.59	0.61	0.65	0.50				
Calf circumference (CALF), cm	0.47	0.80	0.68	0.81	0.86	0.54	0.87	0.49			
Arm length (ARML), cm	0.17	0.28	0.37	0.40	0.54	0.24	0.38	0.19	0.41		
Height, m	−0.06	0.05	0.16	0.26	0.46	0.10	0.33	0.02†	0.36	0.69	
Leg length, cm	−0.14	0.01†	0.02†	0.20	0.29	0.06	0.35	0.02†	0.27	0.50	0.67

* BFP, whole-body fat percentage.

Unless otherwise indicated, all correlations had a P value less than 0.001. †P>0.05; ‡P<0.01. Due to higher frequency of missing data on arm and leg lengths, pairwise was used to handle missing data.

Supplementary Table 4. Correlation matrix (unweighted Pearson's r) between DXA-estimated whole-body fat percentage and more than 350 indices derived using common anthropometrics among adult individuals (≥20 years old) in the development dataset.

Women			***Men***		
**Index***	**r**	**P value**	**Index***	**r**	**P value**
$Height^3$ / (Waist X Weight)	-0.8073	P<0.001	$Height^{0.5}$ / Waist	-0.8481	P<0.001
$Height^2$ / (Waist X $Weight^{0.5}$)	-0.8045	P<0.001	Height / $Waist^{1.5}$	-0.8472	P<0.001
$Height^{2.5}$ / (Waist X Weight)	-0.8042	P<0.001	$Height^{0.5}$ / $Waist^{0.5}$	-0.8459	P<0.001
$Height^2$ / ($Waist^{0.5}$ X $Weight^{0.5}$)	-0.8031	P<0.001	Height / Waist	-0.8448	P<0.001
$Height^{1.5}$ / (Waist X $Weight^{0.5}$)	-0.8018	P<0.001	$Waist^{0.5}$ / $Height^{0.5}$	0.8428	P<0.001
$Height^2$ / ($Waist^{0.5}$ X Weight)	-0.7999	P<0.001	Waist / $Height^{0.5}$	0.8424	P<0.001
$Height^{2.5}$ / (Waist X $Weight^{0.5}$)	-0.7999	P<0.001	Height / $Waist^2$	-0.841	P<0.001
Height / ($ARMC^{0.5}$ X Waist)	-0.798	P<0.001	Waist / Height	0.8386	P<0.001
$Height^2$ / ($Waist^{1.5}$ X $Weight^{0.5}$)	-0.798	P<0.001	$Waist^{1.5}$ / Height	0.8384	P<0.001
$Height^{1.5}$ / (ARMC X Waist)	-0.7976	P<0.001	$Height^2$ / $Waist^2$	-0.8377	P<0.001
$Height^2$ / (Waist X Weight)	-0.7969	P<0.001	$Waist^2$ / Height	0.8292	P<0.001
Height / ($THIGH^{0.5}$ X Waist)	-0.7955	P<0.001	Weight / $Waist^3$	-0.8278	P<0.001
Height / (ARMC X Waist)	-0.7951	P<0.001	Weight / $Waist^4$	-0.8274	P<0.001
$Height^{2.5}$ / Weight	-0.7949	P<0.001	1 / $Waist^{0.5}$	-0.8252	P<0.001
$Height^2$ / (ARMC X Waist)	-0.7945	P<0.001	1 / Waist	-0.824	P<0.001
$Height^3$ / ($Waist^2$ X Weight)	-0.7925	P<0.001	Height / $Waist^3$	-0.8228	P<0.001
Height / (Waist X $Weight^{0.5}$)	-0.7913	P<0.001	$Waist^{0.5}$	0.8224	P<0.001
$Height^2$ / Weight	-0.7899	P<0.001	$Waist^{0.5}$	0.8224	P<0.001
$Height^2$ / ($Waist^2$ X $Weight^{0.5}$)	-0.7898	P<0.001	$Height^2$ / ($Waist^{1.5}$ X $Weight^{0.5}$)	-0.8222	P<0.001
$Height^2$ / ($Waist^{1.5}$ X Weight)	-0.7898	P<0.001	$Height^2$ / ($Waist^2$ X $Weight^{0.5}$)	-0.8193	P<0.001
$Height^3$ / ($Waist^{0.5}$ X $Weight^{0.5}$)	-0.7896	P<0.001	$Waist^3$ / Weight	0.8192	P<0.001
$Height^3$ / (Waist X $Weight^{0.5}$)	-0.7891	P<0.001	$Height^{1.5}$ / Waist	-0.8182	P<0.001
$Height^3$ / Weight	-0.789	P<0.001	Waist / $CALF^{0.5}$	0.8178	P<0.001
Height / $Weight^{0.5}$	-0.788	P<0.001	$Height^2$ / (Waist X $Weight^{0.5}$)	-0.8172	P<0.001
Height / (ARMC X $Waist^{0.5}$)	-0.7876	P<0.001	1 / $Waist^2$	-0.8167	P<0.001
$Height^{0.5}$ / (Waist X Weight)	-0.7875	P<0.001	$Height^{1.5}$ / (Waist X $Weight^{0.5}$)	-0.8142	P<0.001

$Height^{1.5}$ / $Weight^{0.5}$	-0.787	P<0.001	Waist / $Height^{1.5}$	0.8106	P<0.001
$Height^{2}$ / ($THIGH^{0.5}$ X Waist)	-0.7865	P<0.001	Height / ($CALF^{0.5}$ X Waist)	-0.8101	P<0.001
$Height^{0.5}$ / (ARMC X Waist)	-0.7864	P<0.001	$Height^{2.5}$ / (Waist X $Weight^{0.5}$)	-0.8097	P<0.001
$Height^{2}$ / (THIGH X Waist)	-0.7857	P<0.001	$Waist^{2}$ / CALF	0.8087	P<0.001
$Height^{1.5}$ / (Waist X Weight)	-0.7852	P<0.001	Weight / $Waist^{2.5}$	-0.8061	P<0.001
Height / ($CALF^{0.5}$ X Waist)	-0.7845	P<0.001	(Waist X $Weight^{0.5}$) / $Height^{2}$	0.8061	P<0.001
$Height^{2}$ / ($ARMC^{0.5}$ X Waist)	-0.7839	P<0.001	$Waist^{2}$	0.8055	P<0.001
$Height^{1.5}$ / (THIGH X Waist)	-0.7838	P<0.001	$Waist^{2}$	0.8055	P<0.001
$Height^{2}$ / ($Waist^{2}$ X Weight)	-0.7809	P<0.001	Waist / $THIGH^{0.5}$	0.805	P<0.001
$Height^{2}$ / (ARMC X $Waist^{0.5}$)	-0.7746	P<0.001	1 / $Waist^{3}$	-0.8032	P<0.001
$Height^{3.5}$ / Weight	-0.7744	P<0.001	Waist / $ARMC^{0.5}$	0.8008	P<0.001
$Height^{3}$ / (ARMC X Waist)	-0.7743	P<0.001	Height / $Waist^{4}$	-0.8003	P<0.001
Height / (THIGH X Waist)	-0.7743	P<0.001	Height / (Waist X $Weight^{0.5}$)	-0.8003	P<0.001
$Height^{2}$ / ($CALF^{0.5}$ X Waist)	-0.7735	P<0.001	Height / ($ARMC^{0.5}$ X Waist)	-0.7969	P<0.001
$Weight^{0.5}$ / Height	0.7733	P<0.001	$Waist^{2}$ / THIGH	0.7964	P<0.001
$Height^{1.5}$ / Weight	-0.773	P<0.001	Height / ($THIGH^{0.5}$ X Waist)	-0.7962	P<0.001
$Height^{0.5}$ / (Waist X $Weight^{0.5}$)	-0.7729	P<0.001	$Waist^{2.5}$ / Weight	0.7961	P<0.001
$Height^{2}$ / (Waist X $Weight^{1.5}$)	-0.7725	P<0.001	$Height^{3}$ / ($Waist^{2}$ X Weight)	-0.7948	P<0.001
Height / $Waist^{1.5}$	-0.7718	P<0.001	$Height^{3}$ / (Waist X $Weight^{0.5}$)	-0.7934	P<0.001
$Height^{2}$ / (CALF X Waist)	-0.7717	P<0.001	$Waist^{2}$ / ARMC	0.7922	P<0.001
$Weight^{0.5}$ / $Height^{1.5}$	0.7715	P<0.001	$Height^{2}$ / ($CALF^{0.5}$ X Waist)	-0.789	P<0.001
$Height^{1.5}$ / (CALF X Waist)	-0.7706	P<0.001	$Height^{2}$ / ($Waist^{0.5}$ X $Weight^{0.5}$)	-0.7889	P<0.001
$Height^{2}$ / (ARMC X Weight)	-0.7701	P<0.001	$Height^{3}$ / (Waist X Weight)	-0.7882	P<0.001
$Height^{3}$ / (THIGH X Waist)	-0.7701	P<0.001	$Waist^{3}$	0.7857	P<0.001
$Height^{2}$ / $Waist^{2}$	-0.7698	P<0.001	$Height^{2.5}$ / (Waist X Weight)	-0.7844	P<0.001
Height / (Waist X Weight)	-0.7693	P<0.001	1 / $Waist^{4}$	-0.784	P<0.001
Height / $Waist^{2}$	-0.7692	P<0.001	$Waist^{1.5}$ / CALF	0.781	P<0.001
Height / Waist	-0.7689	P<0.001	$Height^{2}$ / ($Waist^{1.5}$ X Weight)	-0.781	P<0.001
$Height^{0.5}$ / Waist	-0.7685	P<0.001	$Height^{2}$ / ($Waist^{2}$ X Weight)	-0.7806	P<0.001
(Waist X $Weight^{0.5}$) / $Height^{2}$	0.7668	P<0.001	$Height^{2}$ / (TRI X Waist)	-0.7774	P<0.001
$Height^{0.5}$ / $Waist^{0.5}$	-0.7659	P<0.001	Height / $Waist^{0.5}$	-0.777	P<0.001
Weight / $Height^{2.5}$	0.7643	P<0.001	$Height^{2}$ / ($ARMC^{0.5}$ X Waist)	-0.7767	P<0.001

$Height^2$ / $Weight^{1.5}$	-0.7619	P<0.001	$Height^2$ / ($THIGH^{0.5}$ X Waist)	-0.7765	P<0.001
Height / (CALF X Waist)	-0.7608	P<0.001	$Height^2$ / Waist	-0.7764	P<0.001
BMI**	0.7605	P<0.001	$Height^{1.5}$ / (TRI X Waist)	-0.7764	P<0.001
Height / $Waist^3$	-0.7589	P<0.001	$Height^{0.5}$ / (Waist X $Weight^{0.5}$)	-0.776	P<0.001
Weight / $Height^3$	0.7579	P<0.001	Height / ($SUB^{0.5}$ X Waist)	-0.7754	P<0.001
$Height^{0.5}$ / (THIGH X Waist)	-0.7572	P<0.001	$Height^2$ / (Waist X Weight)	-0.7747	P<0.001
(ARMC X Waist) / Height	0.7558	P<0.001	$Height^3$ / (TRI X Waist)	-0.7746	P<0.001
$Waist^{0.5}$ / $Height^{0.5}$	0.7549	P<0.001	Height / (TRI X Waist)	-0.7737	P<0.001
(Waist X Weight) / $Height^3$	0.7539	P<0.001	(Waist X Weight) / $Height^3$	0.7728	P<0.001
$Height^{1.5}$ / Waist	-0.7537	P<0.001	$Waist^{0.5}$ / Height	0.7722	P<0.001
$Height^3$ / (TRI X Waist)	-0.7533	P<0.001	$Height^2$ / ($SUB^{0.5}$ X Waist)	-0.7703	P<0.001
$Height^3$ / (CALF X Waist)	-0.7521	P<0.001	$Height^{0.5}$ / (TRI X Waist)	-0.7693	P<0.001
$Height^2$ / $Weight^{0.5}$	-0.7521	P<0.001	(Waist X Weight) / $Height^{2.5}$	0.7688	P<0.001
(Waist X Weight) / $Height^{2.5}$	0.7519	P<0.001	Waist / $Height^2$	0.7667	P<0.001
$Height^2$ / (TRI X Waist)	-0.7514	P<0.001	$Height^3$ / ($Waist^{0.5}$ X $Weight^{0.5}$)	-0.7635	P<0.001
$Height^{0.5}$ / (Weight X Waist)	-0.7497	P<0.001	$Height^{1.5}$ / (CALF X Waist)	-0.7604	P<0.001
1 / Waist	-0.7496	P<0.001	$Height^2$ / (TRI X $Waist^{0.5}$)	-0.7597	P<0.001
1 / $Waist^2$	-0.7491	P<0.001	$Height^{1.5}$ / (Waist X Weight)	-0.7596	P<0.001
$Height^2$ / (THIGH X $Waist^{0.5}$)	-0.7483	P<0.001	$Waist^4$	0.7596	P<0.001
$Height^{1.5}$ / (TRI X Waist)	-0.7478	P<0.001	(Waist X Weight) / $Height^2$	0.7595	P<0.001
Waist / $Height^{0.5}$	0.7478	P<0.001	$Height^2$ / (CALF X Waist)	-0.7586	P<0.001
1 / $Waist^{0.5}$	-0.7473	P<0.001	Height / (TRI X $Waist^{0.5}$)	-0.7568	P<0.001
1 / (Waist X $Weight^{0.5}$)	-0.7471	P<0.001	$Height^2$ / ($Waist^{0.5}$ X Weight)	-0.7553	P<0.001
Waist / Height	0.7467	P<0.001	$Waist^{1.5}$ / THIGH	0.7543	P<0.001
(Waist X Weight) / $Height^2$	0.7459	P<0.001	$Height^{0.5}$ / (Waist X Weight)	-0.7542	P<0.001
Weight / $Height^{1.5}$	0.7458	P<0.001	1 / ($Waist^{0.5}$ X $Height^{0.5}$)	-0.7538	P<0.001
Height / Weight	-0.7448	P<0.001	Height / (CALF X Waist)	-0.7513	P<0.001
$Height^{0.5}$ / $Weight^{0.5}$	-0.7444	P<0.001	(Height X $Waist^2$) /Weight	0.7503	P<0.001
Height / $Waist^4$	-0.744	P<0.001	(Waist X Weight) / $Height^{1.5}$	0.745	P<0.001
Weight / $Height^{3.5}$	0.7431	P<0.001	1 / (Waist X $Weight^{0.5}$)	-0.7433	P<0.001
Height / (THIGH X $Waist^{0.5}$)	-0.7428	P<0.001	(CALF X Waist) / Height	0.742	P<0.001
Height / (TRI X Waist)	-0.7424	P<0.001	(TRI X Waist) / Height	0.7415	P<0.001

(THIGH X Waist) / Height	0.7423	P<0.001	$Height^{1.5}$ / (ARMC X Waist)	-0.7401	P<0.001
1 / $Waist^{3}$	-0.7423	P<0.001	$Waist^{1.5}$ / ARMC	0.7399	P<0.001
$Height^{0.5}$ / (CALF X Waist)	-0.7421	P<0.001	Height / (Waist X Weight)	-0.7394	P<0.001
$Height^{2}$ / ($SUB^{0.5}$ X Waist)	-0.7405	P<0.001	$Height^{2}$ / (ARMC X Waist)	-0.7375	P<0.001
$Waist^{1.5}$ / Height	0.7399	P<0.001	Waist X $Weight^{0.5}$	0.7344	P<0.001
Height / (ARMC X Weight)	-0.7398	P<0.001	Height / (ARMC X Waist)	-0.7343	P<0.001
Height / ($SUB^{0.5}$ X Waist)	-0.7388	P<0.001	Weight / (Height X $Waist^{2}$)	0.7335	P<0.001
(TRI X Waist) / Height	0.7386	P<0.001	$Height^{2}$ / (TRI X Weight)	-0.7332	P<0.001
$Waist^{0.5}$	0.7377	P<0.001	Weight / $Waist^{2}$	-0.7315	P<0.001
$Waist^{0.5}$	0.7377	P<0.001	$Height^{0.5}$ / (CALF X Waist)	-0.7313	P<0.001
Height / ARMC	-0.7365	P<0.001	(ARMC X Waist) / Height	0.73	P<0.001
$Weight^{0.5}$ / $Height^{2}$	0.7365	P<0.001	$Height^{1.5}$ / (THIGH X Waist)	-0.7291	P<0.001
(Waist X Weight) / $Height^{1.5}$	0.7361	P<0.001	Waist X TRI	0.7286	P<0.001
$Height^{0.5}$ / (TRI X Waist)	-0.7353	P<0.001	$Height^{2}$ / (THIGH X Waist)	-0.728	P<0.001
(CALF X Waist) / Height	0.7344	P<0.001	$Height^{3}$ / (CALF X Waist)	-0.7278	P<0.001
Waist X ARMC	0.734	P<0.001	$Height^{2}$ / (Waist X $Weight^{1.5}$)	-0.7276	P<0.001
$Height^{2}$ / $Weight^{2}$	-0.7331	P<0.001	$Height^{1.5}$ / TRI	-0.727	P<0.001
$Height^{0.5}$ / ARMC	-0.7316	P<0.001	$Height^{2}$ / TRI	-0.7268	P<0.001
Waist / $Height^{1.5}$	0.7301	P<0.001	(Waist X Weight) / Height	0.7261	P<0.001
1 / $Waist^{4}$	-0.7296	P<0.001	Height / TRI	-0.7249	P<0.001
$Height^{2}$ / Waist	-0.7279	P<0.001	Waist / $Weight^{0.5}$	0.7231	P<0.001
$Waist^{2}$ / Height	0.7275	P<0.001	$Height^{0.5}$ / TRI	-0.7206	P<0.001
1 / (Waist X Weight)	-0.7266	P<0.001	Height / (THIGH X Waist)	-0.7203	P<0.001
$Height^{2}$ / (TRI X $Waist^{0.5}$)	-0.7261	P<0.001	$Height^{0.5}$ / (ARMC X Waist)	-0.72	P<0.001
$Height^{2}$ / (CALF X $Waist^{0.5}$)	-0.7259	P<0.001	Height / (TRI X Weight)	-0.7193	P<0.001
Height / $Waist^{0.5}$	-0.7248	P<0.001	$Waist^{2}$ / Weight	0.7166	P<0.001
Height / (CALF X $Waist^{0.5}$)	-0.7246	P<0.001	Waist / $Height^{2.5}$	0.7155	P<0.001
$Height^{2}$ / (THIGH X Weight)	-0.7234	P<0.001	$Height^{0.5}$ / (Weight X Waist)	-0.7152	P<0.001
$Height^{1.5}$ / ARMC	-0.7232	P<0.001	1 / TRI	-0.7141	P<0.001
(Waist X Weight) / Height	0.7228	P<0.001	1 / (Height X Waist)	-0.7129	P<0.001
Waist X TRI	0.7223	P<0.001	$Height^{3}$ / (ARMC X Waist)	-0.7113	P<0.001
Weight / $Height^{4}$	0.722	P<0.001	(THIGH X Waist) / Height	0.711	P<0.001

Weight / Height	0.7209	P<0.001	$Height^{2.5}$ / Weight	-0.7102	P<0.001
$Height^{2}$ / (CALF X Weight)	-0.72	P<0.001	Weight / ($Waist^{2}$ X $height^{2}$)	-0.7101	P<0.001
$Height^{2}$ / (TRI X Weight)	-0.7196	P<0.001	Height X Waist	0.7087	P<0.001
ARMC / Height	0.7187	P<0.001	Height / $Weight^{0.5}$	-0.7062	P<0.001
Height / (TRI X $Waist^{0.5}$)	-0.717	P<0.001	Weight / $Height^{2.5}$	0.7061	P<0.001
Waist X $Weight^{0.5}$	0.7161	P<0.001	$Height^{2}$ / (SUB X Waist)	-0.7057	P<0.001
$Waist^{0.5}$ / Height	0.7123	P<0.001	$Height^{1.5}$ / $Weight^{0.5}$	-0.7055	P<0.001
$Waist^{2}$	0.7108	P<0.001	$Height^{1.5}$ / (SUB X Waist)	-0.7055	P<0.001
$Waist^{2}$	0.7108	P<0.001	(SUB X Waist) / Height	0.7045	P<0.001
$Height^{0.5}$ / Weight	-0.7071	P<0.001	$Weight^{0.5}$ / Height	0.7041	P<0.001
1 / ($Waist^{0.5}$ X $Height^{0.5}$)	-0.7069	P<0.001	$Weight^{0.5}$ / $Height^{1.5}$	0.7038	P<0.001
(Weight X Waist) / $Height^{0.5}$	0.7062	P<0.001	Height / (SUB X Waist)	-0.7038	P<0.001
Waist X THIGH	0.7058	P<0.001	(Weight X Waist) / $Height^{0.5}$	0.7036	P<0.001
1 / ARMC	-0.7055	P<0.001	$Height^{2}$ / weight	-0.7033	P<0.001
Waist / $Height^{2}$	0.7027	P<0.001	$Height^{3}$ / Weight	-0.7023	P<0.001
Height / (TRI X Weight)	-0.7009	P<0.001	$Height^{3}$ / (SUB X Waist)	-0.702	P<0.001
$Height^{2}$ / ARMC	-0.6975	P<0.001	$Height^{0.5}$ / (THIGH X Waist)	-0.7016	P<0.001
Waist X CALF	0.6954	P<0.001	$Height^{3}$ / (THIGH X Waist)	-0.7011	P<0.001
Weight / $Waist^{4}$	-0.6946	P<0.001	$Height^{0.5}$ / (SUB X Waist)	-0.7007	P<0.001
Weight / (Height X $Waist^{2}$)	0.6945	P<0.001	BMI**	0.6991	P<0.001
Height / $Weight^{2}$	-0.6924	P<0.001	Weight / $Height^{3}$	0.6987	P<0.001
Weight / $Height^{0.5}$	0.6875	P<0.001	Waist X SUB	0.6958	P<0.001
Waist X Weight	0.6868	P<0.001	Waist X CALF	0.6935	P<0.001
$Weight^{0.5}$ / $Height^{2.5}$	0.6852	P<0.001	Waist X ARMC	0.6925	P<0.001
$Weight^{1.5}$ / Height	0.6851	P<0.001	TRI / $Height^{2}$	0.6905	P<0.001
$Waist^{3}$	0.6849	P<0.001	1 / (Waist X Weight)	-0.6877	P<0.001
$Height^{3}$ / (SUB X Waist)	-0.6839	P<0.001	TRI / Height	0.6872	P<0.001
$Height^{2}$ / (SUB X Waist)	-0.6826	P<0.001	$Height^{3.5}$ / Weight	-0.6828	P<0.001
Height / (THIGH X Weight)	-0.6815	P<0.001	$Height^{1.5}$ / Weight	-0.6814	P<0.001
$Height^{1.5}$ / (SUB X Waist)	-0.6802	P<0.001	Height / (CALF X $Waist^{0.5}$)	-0.6801	P<0.001
ARMC / $Height^{2}$	0.6788	P<0.001	Weight / $Height^{3.5}$	0.6794	P<0.001
$Height^{2}$ / TRI	-0.6782	P<0.001	CALF / Waist	-0.6788	P<0.001

Weight X TRI	0.6781	P<0.001	$Height^{3}$ / Waist	-0.6782	P<0.001
Height / (SUB X Waist)	-0.6765	P<0.001	Waist X Weight	0.6782	P<0.001
$Height^{1.5}$ / TRI	-0.6747	P<0.001	Weight / $Height^{1.5}$	0.6777	P<0.001
$CALF^{0.5}$ / Weight	-0.6745	P<0.001	$Height^{2}$ / (SUB X $Waist^{0.5}$)	-0.6775	P<0.001
Height / (CALF X Weight)	-0.6739	P<0.001	Height / (SUB X $Waist^{0.5}$)	-0.6766	P<0.001
$Height^{0.5}$ / (SUB X Waist)	-0.6715	P<0.001	$Height^{2}$ / (CALF X $Waist^{0.5}$)	-0.6713	P<0.001
Waist / $Height^{2.5}$	0.6694	P<0.001	$Waist^{0.5}$ / $Height^{1.5}$	0.6707	P<0.001
$Height^{2}$ / (SUB X Weight)	-0.6688	P<0.001	Weight X TRI	0.6681	P<0.001
Height / TRI	-0.6686	P<0.001	Height / (ARMC X $Waist^{0.5}$)	-0.6672	P<0.001
CALF / $Weight^{2}$	-0.6673	P<0.001	Waist X THIGH	0.6659	P<0.001
1 / $Weight^{0.5}$	-0.6649	P<0.001	Waist/CALF	0.6656	P<0.001
Weight X $Height^{0.5}$	0.6647	P<0.001	$Height^{2}$ / $Weight^{1.5}$	-0.6654	P<0.001
TRI / $Height^{2}$	0.664	P<0.001	Waist / $Height^{3}$	0.6633	P<0.001
Weight X ARMC	0.6632	P<0.001	$Waist^{0.5}$ / TRI	-0.6608	P<0.001
1 / Weight	-0.6627	P<0.001	$Height^{2}$ / $Weight^{0.5}$	-0.6589	P<0.001
$Height^{3}$ / Waist	-0.6627	P<0.001	$Height^{2}$ / (ARMC X $Waist^{0.5}$)	-0.6577	P<0.001
1 / (Height X Waist)	-0.6611	P<0.001	$Weight^{0.5}$ / $Height^{2}$	0.657	P<0.001
$Height^{0.5}$ / TRI	-0.66	P<0.001	Height X TRI	0.6552	P<0.001
TRI / Height	0.659	P<0.001	$Height^{2}$ / (SUB X Weight)	-0.6546	P<0.001
Weight / $CALF^{0.5}$	0.658	P<0.001	Weight / $Height^{4}$	0.6516	P<0.001
$THIGH^{0.5}$ / Weight	-0.6578	P<0.001	$Height^{0.5}$ / $Weight^{0.5}$	-0.6499	P<0.001
$Weight^{0.5}$	0.6574	P<0.001	Height / Weight	-0.6465	P<0.001
$Height^{2}$ / (SUB X $Waist^{0.5}$)	-0.6564	P<0.001	Weight / Height	0.6443	P<0.001
$Weight^{2}$ / Height	0.6562	P<0.001	Height / (SUB X Weight)	-0.6437	P<0.001
Weight / $Waist^{3}$	-0.6558	P<0.001	TRI / $Weight^{0.5}$	0.6382	P<0.001
Waist / $CALF^{0.5}$	0.6548	P<0.001	Weight X SUB	0.6375	P<0.001
Height / (SUB X Weight)	-0.6547	P<0.001	1 / ($Waist^{0.5}$ X Height)	-0.6354	P<0.001
$Waist^{4}$	0.6534	P<0.001	$Height^{1.5}$ / SUB	-0.6333	P<0.001
(SUB X Waist) / Height	0.6531	P<0.001	Height / SUB	-0.6324	P<0.001
THIGH / $Weight^{2}$	-0.6506	P<0.001	$Height^{2}$ / SUB	-0.6324	P<0.001
1 / ($Weight^{0.5}$ X $Height^{0.5}$)	-0.6505	P<0.001	$Height^{2}$ / $Weight^{2}$	-0.6322	P<0.001
Height / (SUB X $Waist^{0.5}$)	-0.6503	P<0.001	$Height^{0.5}$ / SUB	-0.6296	P<0.001

1 / TRI	-0.6489	P<0.001	Height / (THIGH X $Waist^{0.5}$)	-0.6293	P<0.001
Height X Waist	0.6471	P<0.001	THIGH / Waist	-0.6281	P<0.001
$Waist^{0.5}$ / $Height^{1.5}$	0.6455	P<0.001	$Height^{2}$ / (THIGH X $Waist^{0.5}$)	-0.6258	P<0.001
Weight / $THIGH^{0.5}$	0.6449	P<0.001	Weight / TRI^{2}	-0.6255	P<0.001
1 / $Weight^{2}$	-0.6447	P<0.001	SUB / Height	0.6249	P<0.001
CALF / Weight	-0.6444	P<0.001	1 / SUB	-0.6248	P<0.001
Waist X SUB	0.6433	P<0.001	SUB / $Height^{2}$	0.6211	P<0.001
$Waist^{2}$ / CALF	0.6388	P<0.001	Waist / $TRI^{1.5}$	-0.6166	P<0.001
Waist / $Height^{3}$	0.6339	P<0.001	(Height X Waist) / CALF	0.6165	P<0.001
$Weight^{0.5}$ / $Height^{3}$	0.6309	P<0.001	Waist / THIGH	0.6134	P<0.001
Weight X SUB	0.6257	P<0.001	$Weight^{1.5}$ / Height	0.6103	P<0.001
Weight / CALF	0.6246	P<0.001	$Height^{2}$ / (CALF X Weight)	-0.6075	P<0.001
$Waist^{3}$ / Weight	0.6211	P<0.001	$Height^{2}$ / (ARMC X Weight)	-0.605	P<0.001
$Height^{1.5}$ / THIGH	-0.6191	P<0.001	Weight / THIGH	0.6034	P<0.001
$Weight^{2}$	0.619	P<0.001	THIGH / Weight	-0.6031	P<0.001
$Height^{2}$ / SUB	-0.6141	P<0.001	Height X SUB	0.603	P<0.001
Weight X THIGH	0.6131	P<0.001	$Height^{0.5}$ / Weight	-0.6029	P<0.001
$Height^{1.5}$ / SUB	-0.6117	P<0.001	Weight / $Height^{0.5}$	0.6028	P<0.001
Height / THIGH	-0.6108	P<0.001	Waist / TRI^{2}	-0.6015	P<0.001
$Height^{2}$ / THIGH	-0.6084	P<0.001	Weight / TRI	-0.6001	P<0.001
Height X TRI	0.6083	P<0.001	Weight / $THIGH^{0.5}$	0.5985	P<0.001
Height / SUB	-0.6076	P<0.001	$THIGH^{0.5}$ / Weight	-0.5966	P<0.001
1 / ($Waist^{0.5}$ X Height)	-0.6061	P<0.001	Height X Weight / TRI	-0.5938	P<0.001
Weight X CALF	0.6058	P<0.001	Weight X $Height^{0.5}$	0.5936	P<0.001
Weight / $Waist^{2.5}$	-0.6038	P<0.001	$Weight^{0.5}$ / $Height^{2.5}$	0.5915	P<0.001
THIGH / Height	0.6032	P<0.001	($Height^{2}$ X Waist) / TRI	-0.5902	P<0.001
1 / $Weight^{3}$	-0.6031	P<0.001	(Height X Waist) / TRI	-0.5896	P<0.001
$Height^{0.5}$ / SUB	-0.6019	P<0.001	$Height^{4}$ / Waist	-0.5882	P<0.001
Waist / $THIGH^{0.5}$	0.6006	P<0.001	Height / $Weight^{2}$	-0.587	P<0.001
$Height^{4}$ / Waist	-0.5975	P<0.001	$Weight^{2}$ / Height	0.5864	P<0.001
THIGH / $Height^{2}$	0.5965	P<0.001	THIGH / $Weight^{2}$	-0.5853	P<0.001
1 / SUB	-0.5946	P<0.001	Weight / CALF	0.5829	P<0.001

$Waist^{1.5}$ / CALF	0.594	P<0.001	Weight / $CALF^{0.5}$	0.5826	P<0.001
Height X ARMC	0.593	P<0.001	CALF / Weight	-0.5821	P<0.001
Waist / $ARMC^{0.5}$	0.5917	P<0.001	$CALF^{0.5}$ / Weight	-0.5809	P<0.001
$ARMC^{0.5}$ / Weight	-0.5888	P<0.001	($Height^2$ X Weight) / TRI	-0.5797	P<0.001
SUB / $Height^2$	0.5861	P<0.001	Waist / TRI	-0.579	P<0.001
$Waist^2$ / THIGH	0.5856	P<0.001	($Weight^{0.5}$ X Height) / Waist	-0.5785	P<0.001
Weight / $ARMC^{0.5}$	0.5853	P<0.001	$Waist^{0.5}$ / $Height^2$	0.5777	P<0.001
SUB / Height	0.585	P<0.001	$Height^2$ / (THIGH X Weight)	-0.5743	P<0.001
$Waist^2$ / ARMC	0.583	P<0.001	(Height X Waist) / THIGH	0.5708	P<0.001
$Height^{0.5}$ / THIGH	-0.5803	P<0.001	CALF / $Weight^2$	-0.5695	P<0.001
$Waist^{0.5}$ / $Height^2$	0.5791	P<0.001	Waist / $Height^4$	0.5682	P<0.001
ARMC / $Weight^2$	-0.5789	P<0.001	Height / (ARMC X Weight)	-0.5671	P<0.001
$Weight^2$ X Height	0.5784	P<0.001	ARMC / Waist	-0.5654	P<0.001
$Weight^3$	0.5777	P<0.001	Weight / $ARMC^{0.5}$	0.5646	P<0.001
$Waist^{2.5}$ / Weight	0.577	P<0.001	Waist / ($Weight^{0.5}$ X Height)	0.5638	P<0.001
1 / ($Weight^{0.5}$ X Height)	-0.5728	P<0.001	$ARMC^{0.5}$ / Weight	-0.5625	P<0.001
THIGH / Weight	-0.569	P<0.001	SUB / $Weight^{0.5}$	0.5625	P<0.001
1 / (Weight X Height)	-0.5655	P<0.001	$Weight^{0.5}$	0.561	P<0.001
Waist / $Height^4$	0.5654	P<0.001	TRI^2 / Weight	0.5605	P<0.001
$Waist^{0.5}$ / TRI	-0.5632	P<0.001	Height / (CALF X Weight)	-0.5604	P<0.001
$Height^{1.5}$ / CALF	-0.5626	P<0.001	1 / $Weight^{0.5}$	-0.5598	P<0.001
Weight X Height	0.5613	P<0.001	$Waist^{0.5}$ / SUB	-0.557	P<0.001
Weight / THIGH	0.5608	P<0.001	TRI / Weight	0.5558	P<0.001
CALF / Height	0.56	P<0.001	1 / Weight	-0.5551	P<0.001
Height / CALF	-0.557	P<0.001	ARMC / $Weight^2$	-0.552	P<0.001
Height X SUB	0.5568	P<0.001	1 / ($Weight^{0.5}$ X $Height^{0.5}$)	-0.5518	P<0.001
$Height^2$ / CALF	-0.5457	P<0.001	Waist/ARMC	0.5502	P<0.001
CALF / $Height^2$	0.5428	P<0.001	$Weight^2$	0.5419	P<0.001
Weight / $Waist^{0.5}$	0.5421	P<0.001	TRI / Waist	0.5415	P<0.001
TRI / $Weight^{0.5}$	0.5361	P<0.001	1 / $Weight^2$	-0.5373	P<0.001
1 / THIGH	-0.5309	P<0.001	Height / (THIGH X Weight)	-0.5319	P<0.001
1 / $Weight^4$	-0.5278	P<0.001	$Weight^{0.5}$ / $Height^3$	0.5268	P<0.001

$Weight^4$	0.5274	P<0.001	Weight X ARMC	0.5211	P<0.001
$Waist^{0.5}$ / SUB	-0.5272	P<0.001	$Weight^3$	0.515	P<0.001
$Height^{0.5}$ / CALF	-0.5221	P<0.001	Weight / ARMC	0.5115	P<0.001
$Waist^{0.5}$ / $Height^{2.5}$	0.5219	P<0.001	ARMC / Weight	-0.5091	P<0.001
Weight X $Height^{1.5}$	0.5179	P<0.001	1 / $Weight^3$	-0.5086	P<0.001
Weight / TRI^2	-0.5137	P<0.001	SUB^2 / Weight	0.508	P<0.001
$Waist^{1.5}$ / THIGH	0.5088	P<0.001	Weight X CALF	0.5078	P<0.001
Waist / TRI^2	-0.5088	P<0.001	$Waist^{0.5}$ / $Height^{2.5}$	0.5026	P<0.001
Waist / $TRI^{1.5}$	-0.5074	P<0.001	Weight / $Waist^{1.5}$	-0.4986	P<0.001
(Height X Weight) / CALF	0.503	P<0.001	$Weight^2$ X Height	0.4967	P<0.001
TRI^2 / Weight	0.4928	P<0.001	Weight / SUB	-0.4893	P<0.001
Weight / $Waist^2$	-0.4878	P<0.001	(Height X Waist) / ARMC	0.4882	P<0.001
SUB / $Weight^{0.5}$	0.4874	P<0.001	($Height^2$ X Waist) / CALF	0.4882	P<0.001
Weight / SUB^2	-0.4815	P<0.001	Weight X THIGH	0.4864	P<0.001
$Weight^{0.5}$ X Height	0.4813	P<0.001	Height X Weight / SUB	-0.4827	P<0.001
Waist / $SUB^{1.5}$	-0.4794	P<0.001	Height / ARMC	-0.4801	P<0.001
Weight X $Height^2$	0.4764	P<0.001	$Waist^{1.5}$ / Weight	0.4793	P<0.001
Waist / $Weight^{0.5}$	0.4762	P<0.001	ARMC / Height	0.4788	P<0.001
Waist / SUB^2	-0.4753	P<0.001	$Weight^4$	0.4767	P<0.001
CALF / Waist	-0.475	P<0.001	$Height^{1.5}$ / ARMC	-0.4753	P<0.001
$Waist^{1.5}$ / ARMC	0.4745	P<0.001	1 / ($Weight^{0.5}$ X Height)	-0.4745	P<0.001
$Waist^{0.5}$ / $Height^3$	0.4745	P<0.001	Waist / $SUB^{1.5}$	-0.4739	P<0.001
$Waist^2$ / Weight	0.469	P<0.001	($Height^2$ X Weight) / SUB	-0.4701	P<0.001
($Height^2$ X Waist) / TRI	-0.4684	P<0.001	1 / $Weight^4$	-0.4696	P<0.001
(Height X $Waist^2$) /Weight	0.467	P<0.001	Weight X Height	0.4671	P<0.001
1 / CALF	-0.463	P<0.001	($Height^2$ X Waist) / SUB	-0.4666	P<0.001
Waist/CALF	0.4593	P<0.001	(Height X Waist) / SUB	-0.4663	P<0.001
(Height X Waist) / TRI	-0.4548	P<0.001	$Height^{0.5}$ / ARMC	-0.465	P<0.001
($Height^2$ X Waist) / SUB	-0.4546	P<0.001	$Waist^{1.5}$ / TRI	-0.4632	P<0.001
$Waist^{0.5}$ / ARMC	-0.4492	P<0.001	($Height^2$ X Waist) / THIGH	0.4626	P<0.001
Waist / $ARMC^2$	-0.4454	P<0.001	1 / (Weight X Height)	-0.4606	P<0.001
(Height X Waist) / SUB	-0.4452	P<0.001	Waist / SUB	-0.4582	P<0.001

SUB^2 / Weight	0.4387	P<0.001	SUB / Weight	0.4569	P<0.001
Weight / (Waist X $height^2$)	0.4338	P<0.001	$Height^2$ / ARMC	-0.4561	P<0.001
($Weight^{0.5}$ X Height) / Waist	-0.4338	P<0.001	Height X Weight / THIGH	0.4543	P<0.001
Waist / TRI	-0.4311	P<0.001	ARMC / $Height^2$	0.4517	P<0.001
Waist / SUB	-0.4289	P<0.001	SUB / Waist	0.4509	P<0.001
Height X Weight / THIGH	0.4288	P<0.001	CALF / Height	0.445	P<0.001
Weight / ($Waist^2$ X $height^2$)	-0.4287	P<0.001	$Waist^{0.5}$ / $Height^3$	0.4437	P<0.001
Height X THIGH	0.4205	P<0.001	(Height X Weight) / CALF	0.4423	P<0.001
Waist / ($Weight^{0.5}$ X Height)	0.4194	P<0.001	Weight / SUB^2	-0.4409	P<0.001
(Height X Waist) / CALF	0.4152	P<0.001	Height / CALF	-0.4397	P<0.001
SUB / Waist	0.4071	P<0.001	$Height^{1.5}$ / CALF	-0.437	P<0.001
$Weight^{0.5}$ X $Height^{1.5}$	0.4045	P<0.001	1 / ARMC	-0.4307	P<0.001
TRI / Waist	0.4015	P<0.001	Waist / $CALF^{1.5}$	0.4279	P<0.001
Weight / $ARMC^2$	-0.3972	P<0.001	Weight X $Height^{1.5}$	0.4257	P<0.001
($Height^2$ X Weight) / SUB	-0.3951	P<0.001	$Height^2$ / CALF	-0.4142	P<0.001
Height X Weight / SUB	-0.3938	P<0.001	CALF / $Height^2$	0.4139	P<0.001
($Height^2$ X Weight) / TRI	-0.3925	P<0.001	$Height^{0.5}$ / CALF	-0.4134	P<0.001
Height X Weight / TRI	-0.3909	P<0.001	Waist / SUB^2	-0.4053	P<0.001
ARMC / $Weight^{0.5}$	0.3907	P<0.001	$Weight^{0.5}$ X Height	0.3901	P<0.001
($Height^2$ X Weight) / CALF	0.3891	P<0.001	Weight X $Height^2$	0.3877	P<0.001
Weight / SUB	-0.3866	P<0.001	THIGH / Height	0.3823	P<0.001
$ARMC^2$ / Weight	0.3861	P<0.001	$Height^{1.5}$ / THIGH	-0.3773	P<0.001
Weight / TRI	-0.3803	P<0.001	Waist / $THIGH^{1.5}$	0.376	P<0.001
Weight / ARMC	0.3783	P<0.001	Height / THIGH	-0.3734	P<0.001
ARMC / Weight	-0.3695	P<0.001	Weight / $Waist^{0.5}$	0.3722	P<0.001
Weight / (Height X Waist)	0.3617	P<0.001	($Height^2$ X Waist) / ARMC	0.3711	P<0.001
SUB / Weight	0.3423	P<0.001	THIGH / $Height^2$	0.3696	P<0.001
Height X CALF	0.3422	P<0.001	$Height^2$ / THIGH	-0.366	P<0.001
TRI / Weight	0.3413	P<0.001	1 / CALF	-0.3635	P<0.001
$Weight^{0.5}$ X $Height^2$	0.3397	P<0.001	(Height X Weight) / ARMC	0.3626	P<0.001
($Height^2$ X Waist) / CALF	0.326	P<0.001	$Height^{0.5}$ / THIGH	-0.3505	P<0.001
THIGH / Waist	-0.3189	P<0.001	($Height^2$ X Weight) / THIGH	0.3391	P<0.001

(Height^2 X Weight) / THIGH	0.3136	P<0.001	Height X ARMC	0.335	P<0.001
Waist / THIGH	0.3119	P<0.001	(Height^2 X Weight) / CALF	0.3321	P<0.001
Waist$^{1.5}$ / SUB	-0.2966	P<0.001	Weight$^{0.5}$ X Height$^{1.5}$	0.3248	P<0.001
CALF / Weight$^{0.5}$	-0.2871	P<0.001	Waist$^{1.5}$ / SUB	-0.3244	P<0.001
Weight$^{0.5}$ X Height$^{2.5}$	0.2858	P<0.001	Waist / TRI$^{0.5}$	-0.321	P<0.001
CALF^2 / Weight	-0.2851	P<0.001	Waist^2 / TRI	-0.3157	P<0.001
Weight / CALF^2	0.28	P<0.001	CALF / Weight$^{0.5}$	-0.3151	P<0.001
(Height X Waist) / THIGH	0.2675	P<0.001	CALF^2 / Weight	-0.3138	P<0.001
Waist / ARMC$^{1.5}$	-0.2629	P<0.001	1 / THIGH	-0.312	P<0.001
Waist$^{1.5}$ / TRI	-0.2562	P<0.001	Weight / CALF^2	0.3114	P<0.001
(Height X Weight) / ARMC	0.2462	P<0.001	Weight / TRI$^{0.5}$	-0.3062	P<0.001
Weight / Waist	0.2425	P<0.001	TRI$^{0.5}$ / Weight	0.2922	P<0.001
Weight$^{0.5}$ X Height^3	0.2411	P<0.001	THIGH / Weight$^{0.5}$	-0.2885	P<0.001
Waist / CALF$^{1.5}$	0.232	P<0.001	THIGH^2 / Weight	-0.2874	P<0.001
Waist / Weight	-0.2305	P<0.001	Weight / THIGH^2	0.2828	P<0.001
Weight / Waist$^{1.5}$	-0.2124	P<0.001	TRI / Weight^2	0.2749	P<0.001
Waist$^{1.5}$ / Weight	0.2071	P<0.001	Weight$^{0.5}$ X Height^2	0.2723	P<0.001
(Height^2 X Waist) / THIGH	0.195	P<0.001	Height X CALF	0.2601	P<0.001
(Height^2 X Weight) / ARMC	0.1565	P<0.001	(Height^2 X Weight) / ARMC	0.2599	P<0.001
Height^2	-0.15	P<0.001	Waist / ARMC$^{1.5}$	0.2371	P<0.001
Height^3	-0.15	P<0.001	Height X THIGH	0.2356	P<0.001
Height^4	-0.1498	P<0.001	Waist$^{0.5}$ / CALF	0.2348	P<0.001
Height$^{0.5}$	-0.1497	P<0.001	Waist / CALF^2	0.2331	P<0.001
1 / Height$^{0.5}$	0.1493	P<0.001	Weight$^{0.5}$ X Height$^{2.5}$	0.2299	P<0.001
1 / Height	0.149	P<0.001	Waist$^{0.5}$ / THIGH	0.2075	P<0.001
1 / Height^2	0.1483	P<0.001	Waist / THIGH^2	0.2045	P<0.001
1 / Height^3	0.1474	P<0.001	Weight$^{0.5}$ X Height^3	0.1954	P<0.001
1 / Height^4	0.1464	P<0.001	Waist / SUB$^{0.5}$	-0.1743	P<0.001
Waist/ARMC	0.1458	P<0.001	Weight / SUB$^{0.5}$	-0.1705	P<0.001
(Height X Weight) / Waist	0.1454	P<0.001	Waist^2 / SUB	-0.1617	P<0.001
Waist / SUB$^{0.5}$	-0.1427	P<0.001	SUB$^{0.5}$ / Weight	0.1604	P<0.001
ARMC / Waist	-0.1404	P<0.001	SUB / Weight^2	0.1506	P<0.001

$Waist^2$ / SUB	-0.1389	P<0.001	$Height^4$	-0.0926	P<0.001
Waist / (Weight X Height)	-0.1345	P<0.001	$Height^3$	-0.0917	P<0.001
$Waist^{0.5}$ / THIGH	-0.1217	P<0.001	$Height^2$	-0.0908	P<0.001
Weight / $TRI^{0.5}$	0.1202	P<0.001	$Height^{0.5}$	-0.0893	P<0.001
Waist / $THIGH^2$	-0.114	P<0.001	1 / $Height^{0.5}$	0.0881	P<0.001
$TRI^{0.5}$ / Weight	-0.1091	P<0.001	1 / Height	0.0875	P<0.001
TRI / $Weight^2$	-0.1021	P<0.001	1 / $Height^2$	0.0863	P<0.001
(Height X Waist) / ARMC	0.0675	P<0.001	1 / $Height^3$	0.0849	P<0.001
$Waist^2$ / TRI	-0.0659	P<0.001	1 / $Height^4$	0.0835	P<0.001
Waist / $TRI^{0.5}$	-0.0642	P<0.001	Weight / (Waist X $height^2$)	0.0737	P<0.001
Waist / $CALF^2$	0.0613	P<0.001	$ARMC^2$ / Weight	-0.0556	P<0.001
$Waist^{0.5}$ / CALF	0.057	P<0.001	ARMC / $Weight^{0.5}$	-0.0515	P=0.001
Waist / $THIGH^{1.5}$	0.0485	P=0.003	(Height X Weight) / Waist	-0.0468	P=0.002
Weight / $SUB^{0.5}$	0.0243	P=0.132	Waist / (Weight X Height)	0.0422	P=0.006
$SUB^{0.5}$ / Weight	-0.0188	P=0.244	$Waist^{0.5}$ / ARMC	0.041	P=0.007
SUB / $Weight^2$	-0.0165	P=0.307	Weight / $ARMC^2$	0.0378	P=0.014
Weight / $THIGH^2$	0.0154	P=0.341	Waist / $ARMC^2$	0.035	P=0.022
$THIGH^2$ / Weight	0.0043	P=0.792	Weight / Waist	-0.0234	P=0.127
THIGH / $Weight^{0.5}$	-0.0007	P=0.964	Waist / Weight	0.0204	P=0.181
($Height^2$ X Waist) / ARMC	-0.0003	P=0.983	Weight / (Height X Waist)	0.02	P=0.191

*ARMC, arm circumference; CALF, calf circumference; SUB, subscapular skinfold; THIGH, thigh circumference; TRI, triceps skinfold.

** Correlation between body fat percentage and BMI is shown for comparison purposes.

Supplementary Table 5. Prediction of DXA-estimated whole-body fat percentage by sex and ethnicity among adult participants in NHANES 1999-2004.*

	**All**	**Mexican-American**	**European-American**	**African-American**
**Women**	N=6,261	N=1,399	N=3,107	N=1,244
**R^2 (95% CI) †**				
BMI ‡	0.63 (0.62 to 0.65)	0.60 (0.57 to 0.63)	0.65 (0.63 to 0.67)	0.65 (0.63 to 0.67)
1/BMI equation §	0.71 (0.69 to 0.72)	0.65 (0.61 to 0.69)	0.72 (0.70 to 0.74)	0.73 (0.71 to 0.75)
Height/waist equation ¶	0.66 (0.64 to 0.68)	0.59 (0.55 to 0.63)	0.67 (0.65 to 0.70)	0.66 (0.63 to 0.69)
Waist/height equation #	0.63 (0.61 to 0.64)	0.56 (0.53 to 0.59)	0.64 (0.62 to 0.66)	0.62 (0.59 to 0.65)
(√Height)/waist equation ||	0.66 (0.64 to 0.68)	0.59 (0.55 to 0.64)	0.67 (0.65 to 0.70)	0.66 (0.63 to 0.69)
$Height^2$/(waist X √weight) equation **	0.71 (0.69 to 0.73)	0.65 (0.61 to 0.68)	0.73 (0.71 to 0.75)	0.71 (0.69 to 0.74)
$Height^3$/(waist X weight) equation ††	0.72 (0.70 to 0.73)	0.65 (0.62 to 0.69)	0.73 (0.71 to 0.75)	0.72 (0.70 to 0.74)
**RMSE (95% CI)** §§				
BMI	4.09 (4.00 to 4.19)	3.70 (3.51 to 3.88)	4.11 (3.99 to 4.22)	4.05 (3.87 to 4.23)
1/BMI equation	3.68 (3.59 to 3.77)	3.47 (3.29 to 3.65)	3.66 (3.55 to 3.78)	3.56 (3.42 to 3.71)
Height/waist equation	3.95 (3.84 to 4.07)	3.73 (3.56 to 3.91)	3.97 (3.81 to 4.13)	4.01 (3.83 to 4.18)
Waist/height equation	4.15 (4.03 to 4.26)	3.87 (3.71 to 4.03)	4.17 (4.03 to 4.32)	4.23 (4.05 to 4.41)
(√Height)/waist equation	3.94 (3.82 to 4.06)	3.72 (3.54 to 3.90)	3.96 (3.81 to 4.12)	4.01 (3.83 to 4.18)
$Height^2$/(waist X √weight) equation	3.64 (3.54 to 3.74)	3.48 (3.31 to 3.65)	3.63 (3.49 to 3.77)	3.66 (3.51 to 3.82)
$Height^3$/(waist X weight) equation	3.61 (3.51 to 3.71)	3.45 (3.28 to 3.62)	3.59 (3.47 to 3.72)	3.62 (3.47 to 3.77)
**Men**	N=6,320	N=1,439	N=3,194	N=1,202
**R^2 (95% CI)**				
BMI	0.57 (0.55 to 0.58)	0.64 (0.60 to 0.68)	0.57 (0.55 to 0.60)	0.63 (0.59 to 0.66)
1/BMI equation	0.59 (0.57 to 0.61)	0.65 (0.62 to 0.69)	0.60 (0.57 to 0.62)	0.63 (0.60 to 0.67)
Height/waist equation	0.75 (0.74 to 0.76)	0.72 (0.69 to 0.75)	0.75 (0.74 to 0.77)	0.80 (0.77 to 0.82)
Waist/height equation	0.73 (0.71 to 0.74)	0.71 (0.68 to 0.74)	0.73 (0.71 to 0.75)	0.78 (0.76 to 0.80)
(√Height)/waist equation	0.76 (0.74 to 0.77)	0.74 (0.71 to 0.77)	0.76 (0.74 to 0.78)	0.80 (0.77 to 0.82)
$Height^2$/(waist X √weight) equation	0.71 (0.70 to 0.73)	0.71 (0.68 to 0.74)	0.72 (0.70 to 0.73)	0.75 (0.73 to 0.78)
$Height^3$/(waist X weight) equation	0.67 (0.66 to 0.69)	0.69 (0.66 to 0.72)	0.68 (0.66 to 0.70)	0.71 (0.68 to 0.74)
**RMSE (95% CI)**				

BMI	4.02 (3.93 to 4.12)	3.26 (3.13 to 3.38)	3.93 (3.81 to 4.05)	4.18 (4.01 to 4.34)
1/BMI equation	3.91 (3.82 to 4.00)	3.19 (3.06 to 3.33)	3.82 (3.71 to 3.92)	4.14 (3.97 to 4.32)
Height/waist equation	3.04 (2.98 to 3.10)	2.86 (2.72 to 3.01)	2.99 (2.92 to 3.07)	3.08 (2.94 to 3.23)
Waist/height equation	3.18 (3.13 to 3.23)	2.90 (2.77 to 3.03)	3.14 (3.07 to 3.22)	3.20 (3.05 to 3.35)
(√Height)/waist equation	3.01 (2.95 to 3.07)	2.76 (2.63 to 2.89)	2.95 (2.88 to 3.03)	3.08 (2.93 to 3.23)
Height^2/(waist X √weight) equation	3.27 (3.21 to 3.33)	2.90 (2.76 to 3.04)	3.21 (3.13 to 3.29)	3.40 (3.25 to 3.55)
Height^3/(waist X weight) equation	3.49 (3.42 to 3.56)	3.01 (2.86 to 3.15)	3.42 (3.33 to 3.50)	3.70 (3.54 to 3.85)

* Values represent pooled weighted estimates with bootstrapped 95% confidence intervals (95% CI) from DXA imputed data.
† R^2, coefficient of determination.
‡ BMI, body mass index.
§ 1/BMI equation: 54 – (700 X (1/BMI)) + (12 X sex).
¶ Height/waist equation (Relative fat mass, RFM): 64 – (20 X height/waist) + (12 X sex).
# Waist/height equation: – 5 + 58 X (waist/height) + (11 X sex)
|| (√Height)/waist equation: 65 – (27 X (√height)/waist) + (12 X sex).
** Height^2/(waist X √weight) equation: 50 – (63 X (height^2)/(waist X √body weight)) + (12 X sex).
†† Height^3/(waist X weight) equation: 44 – (230 X (height^3)/(waist X body weight)) + (12 X sex).
§§ RMSE, root mean squared error.
For all models, sex= 0 for male and 1 for female. Height and waist (circumference) are expressed in meters; body weight in kilograms.

Supplementary Table 6. Prediction of whole-body fat percentage in NHANES 1999-2004, by age category*.

	**20-39 years old**	**40-59 years old**	**≥60 years old**
**Women**	N=1,989	N=1,998	N=2,274
**R^2 (95% CI) †**			
BMI	0.70 (0.68 to 0.72)	0.64 (0.61 to 0.66)	0.58 (0.55 to 0.60)
1/BMI equation	0.76 (0.74 to 0.77)	0.70 (0.68 to 0.73)	0.64 (0.61 to 0.67)
Height/waist equation	0.70 (0.68 to 0.73)	0.64 (0.61 to 0.67)	0.49 (0.46 to 0.53)
Waist/height equation	0.67 (0.65 to 0.70)	0.61 (0.58 to 0.64)	0.48 (0.44 to 0.52)
(√Height)/waist equation	0.70 (0.68 to 0.73)	0.64 (0.61 to 0.67)	0.51 (0.47 to 0.54)
Height^2/(waist X √weight) equation	0.75 (0.73 to 0.77)	0.69 (0.67 to 0.72)	0.59 (0.55 to 0.62)
Height^3/(waist X weight) equation	0.75 (0.73 to 0.77)	0.70 (0.67 to 0.73)	0.61 (0.58 to 0.64)
**RMSE (95% CI) ‡**			
BMI	3.91 (3.76 to 4.05)	3.92 (3.75 to 4.09)	3.66 (3.50 to 3.82)
1/BMI equation	3.55 (3.43 to 3.67)	3.54 (3.37 to 3.70)	3.38 (3.25 to 3.50)
Height/waist equation	3.91 (3.76 to 4.06)	3.89 (3.71 to 4.08)	4.01 (3.85 to 4.17)
Waist/height equation	4.12 (3.97 to 4.27)	4.07 (3.89 to 4.25)	4.07 (3.89 to 4.25)
(√Height)/waist equation	3.92 (3.76 to 4.07)	3.88 (3.70 to 4.07)	3.96 (3.80 to 4.12)
Height^2/(waist X √weight) equation	3.60 (3.47 to 3.73)	3.60 (3.42 to 3.77)	3.62 (3.49 to 3.76)
Height^3/(waist X weight) equation	3.60 (3.47 to 3.73)	3.55 (3.38 to 3.72)	3.51 (3.38 to 3.63)
**Men**	N=2,130	N=1,976	N=2,214
**R^2 (95% CI)**			
BMI	0.62 (0.60 to 0.65)	0.56 (0.53 to 0.60)	0.53 (0.50 to 0.56)
1/BMI equation	0.64 (0.62 to 0.67)	0.58 (0.54 to 0.62)	0.53 (0.50 to 0.56)
Height/waist equation	0.78 (0.76 to 0.79)	0.70 (0.67 to 0.73)	0.63 (0.60 to 0.67)
Waist/height equation	0.76 (0.74 to 0.77)	0.68 (0.65 to 0.70)	0.63 (0.60 to 0.66)
(√Height)/waist equation	0.79 (0.77 to 0.80)	0.71 (0.68 to 0.74)	0.64 (0.61 to 0.67)
Height^2/(waist X √weight) equation	0.74 (0.72 to 0.76)	0.67 (0.64 to 0.70)	0.61 (0.58 to 0.64)
Height^3/(waist X weight) equation	0.70 (0.68 to 0.72)	0.63 (0.60 to 0.67)	0.58 (0.55 to 0.61)
**RMSE (95% CI)**			
BMI	3.95 (3.80 to 4.10)	3.56 (3.42 to 3.71)	3.61 (3.50 to 3.72)
1/BMI equation	3.86 (3.72 to 4.00)	3.51 (3.36 to 3.66)	3.60 (3.49 to 3.70)
Height/waist equation	3.04 (2.94 to 3.13)	2.95 (2.85 to 3.06)	3.17 (3.07 to 3.27)
Waist/height equation	3.18 (3.08 to 3.29)	3.06 (2.97 to 3.16)	3.19 (3.08 to 3.29)
(√Height)/waist equation	2.98 (2.88 to 3.09)	2.91 (2.80 to 3.02)	3.15 (3.06 to 3.24)
Height^2/(waist X √weight) equation	3.29 (3.18 to 3.41)	3.10 (2.99 to 3.22)	3.27 (3.17 to 3.37)
Height^3/(waist X weight) equation	3.53 (3.40 to 3.66)	3.26 (3.14 to 3.38)	3.40 (3.30 to 3.49)

* Values represent pooled weighted estimates with bootstrapped 95% confidence intervals (95% CI) from DXA imputed data.
† R^2, coefficient of determination.
‡ RMSE, root mean squared error.

Supplementary Table 7. Raw equations developed for the prediction of DXA-estimated whole-body fat percentage among participants (≥20 years old) in NHANES 1999-2004.

**1/BMI**	
Equation for women	65.314 – (678.528 X (1/BMI))
Equation for men	53.847 – (696.428 X (1/BMI))
Equation for women and men	53.436 – (685.361 X (1/BMI)) + (12.134 X sex)
**Height/waist**	
Equation for women	73.071 – (18.554 X height/waist)
Equation for men	64.980 – (20.469 X height/waist)
Equation for women and men	62.979 – (19.361 X height/waist) + (11.536 X sex)
**Waist/height**	
Equation for women	8.391 + 54.773 X (waist/height)
Equation for men	-7.532 + 62.878 X (waist/height)
Equation for women and men	-4.820 + 58.078 X (waist/height) + (11.311 X sex)
**(√Height)/waist**	
Equation for women	73.723 – (24.092 X (√height)/waist)
Equation for men	65.404 – (27.471 X (√height)/waist)
Equation for women and men	62.675 – (254.666 X (√height)/waist) + (12.979 X sex)
**Height^2/(waist X √weight)**	
Equation for women	61.041 – (60.461 X (height^2)/(waist X √weight))
Equation for men	50.580 – (60.461 X (height^2)/(waist X √weight))
Equation for women and men	49.765 – (62.062 X (height^2)/(waist X √weight)) + (11.836 X sex)
**Height^3/(waist X weight)**	
Equation for women	55.134 – (220.092 X (height^3)/(waist X weight))
Equation for men	43.804 – (230.309 X (height^3)/(waist X weight))
Equation for women and men	43.384 – (224.188 X (height^3)/(waist X weight)) + (12.034 X sex)

For all models, height and waist (circumference) are expressed in meters; weight in kilograms.

Supplementary Table 8. Concordance correlation coefficients (rho_c) between predicted and DXA-estimated body fat percentage by sex and ethnicity in NHANES 1999-2004.*****

	**All**	***Mexican-American***	***European-American***	***African-American***
**Women**				
BMI	0.32	0.27	0.32	0.44
1/BMI equation	0.83	0.80	0.84	0.81
Height/waist equation	0.80	0.76	0.81	0.78
(√Height)/waist equation	0.80	0.76	0.81	0.81
$Height^2$/(waist X √weight) equation	0.84	0.80	0.84	0.81
$Height^3$/(waist X weight) equation	0.83	0.80	0.84	0.81
**Men**				
BMI	0.75	0.79	0.75	0.74
1/BMI equation	0.74	0.78	0.75	0.73
Height/waist equation	0.86	0.83	0.85	0.88
(√Height)/waist equation	0.86	0.85	0.86	0.86
$Height^2$/(waist X √weight) equation	0.83	0.83	0.83	0.85
$Height^3$/(waist X weight) equation	0.80	0.81	0.80	0.81

* Coefficients represent pooled weighted estimates.

Supplementary Table 9. Performance of selected regression model equations for the estimation of body fat percentage in NHANES 1999-2004.*

	All	Mexican-American	European-American	African-American
**Women**				
**Bias (95% CI)†**				
BMI	−11.9 (−12.2 to −11.7)	−12.2 (−12.7 to −11.7)	−12.2 (−12.4 to −11.9)	−9.7 (−10.1 to −9.3)
1/BMI equation	−0.2 (−0.4 to 0.0)	−0.2 (−0.4 to 0.1)	−0.4 (−0.6 to −0.2)	1.5 (1.2 to 1.8)
Height/waist equation	0.4 (0.2 to 0.6)	0.8 (0.5 to 1.2)	0.1 (−0.1 to 0.4)	1.3 (1.0 to 1.7)
(√Height)/waist equation	−0.7 (−1.0 to −0.5)	−0.6 (−1.0 to −0.2)	−0.9 (−1.1 to −0.7)	0.5 (0.1 to 0.9)
$Height^2$/(waist X √weight) equation	0.1 (−0.1 to 0.3)	0.4 (0.0 to 0.7)	−0.2 (−0.4 to 0.0)	1.4 (1.0 to 1.7)
$Height^3$/(waist X weight) equation	−0.1 (−0.3 to 0.0)	0.1 (−0.2 to 0.4)	−0.4 (−0.6 to −0.2)	1.1 (0.8 to 1.5)
**Accuracy (95% CI)‡**				
BMI	15.4 (14.0 to 16.8)	17.1 (14.4 to 19.9)	12.7 (11.2 to 14.2)	33.1 (30.2 to 36.0)
1/BMI equation	94.8 (94.0 to 95.6)	96.2 (95.0 to 97.5)	94.8 (93.8 to 95.8)	92.1 (90.7 to 93.5)
Height/waist equation	93.3 (92.2 to 94.4)	94.5 (92.9 to 96.2)	93.2 (91.7 to 94.6)	91.6 (90.2 to 93.0)
(√Height)/waist equation	94.2 (93.4 to 95.0)	95.7 (94.5 to 96.9)	94.1 (92.9 to 95.2)	93.1 (91.8 to 94.3)
$Height^2$/(waist X √weight) equation	95.1 (94.2 to 96.0)	96.0 (94.6 to 97.4)	95.3 (94.1 to 96.4)	92.5 (91.1 to 93.8)
$Height^3$/(waist X weight) equation	95.3 (94.5 to 96.1)	96.5 (95.4 to 97.6)	95.4 (94.4 to 96.4)	92.8 (91.5 to 94.2)
**Precision (95% CI)§**				
BMI	5.6 (5.4 to 5.7)	5.4 (5.1 to 5.7)	5.4 (5.2 to 5.6)	6.0 (5.7 to 6.3)
1/BMI equation	4.7 (4.5 to 4.9)	4.5 (4.1 to 4.8)	4.7 (4.5 to 5.0)	4.7 (4.4 to 5.0)
Height/waist equation	5.3 (5.1 to 5.5)	4.8 (4.3 to 5.2)	5.4 (5.1 to 5.6)	5.3 (4.9 to 5.6)
(√Height)/waist equation	5.3 (5.1 to 5.5)	5.0 (4.6 to 5.5)	5.4 (5.2 to 5.6)	5.3 (4.9 to 5.7)
$Height^2$/(waist X √weight) equation	4.9 (4.7 to 5.1)	4.4 (4.1 to 4.7)	4.9 (4.7 to 5.1)	4.9 (4.6 to 5.2)
$Height^3$/(waist X weight) equation	4.8 (4.6 to 5.0)	4.4 (4.1 to 4.7)	4.8 (4.6 to 5.0)	4.9 (4.6 to 5.2)
**Men**				
**Bias (95% CI)**				
BMI	0.0 (−0.2 to 0.2)	−0.1 (−0.4 to 0.2)	−0.4 (−0.6 to −0.1)	2.5 (2.2 to 2.9)
1/BMI equation	0.1 (−0.1 to 0.3)	0.2 (0.0 to 0.5)	−0.3 (−0.5 to 0.0)	2.2 (1.9 to 2.5)
Height/waist equation	0.0 (−0.1 to 0.1)	0.5 (0.2 to 0.7)	−0.2 (−0.3 to −0.1)	0.8 (0.6 to 1.1)
(√Height)/waist equation	0.4 (0.2 to 0.5)	0.1 (−0.1 to 0.4)	0.3 (0.2 to 0.5)	1.2 (1.0 to 1.5)
$Height^2$/(waist X √weight) equation	0.0 (−0.1 to 0.2)	0.3 (0.1 to 0.6)	−0.2 (−0.4 to 0.0)	1.3 (1.1 to 1.6)

Height³/(waist X weight) equation	0.3 (0.2 to 0.5)	0.7 (0.4 to 0.9)	0.1 (−0.2 to 0.3)	1.8 (1.5 to 2.1)
**Accuracy (95% CI)**				
BMI	82.0 (80.7 to 83.2)	90.5 (88.6 to 92.5)	83.6 (82.2 to 85.1)	66.6 (63.8 to 69.3)
1/BMI equation	83.6 (82.3 to 84.8)	90.9 (89.1 to 92.7)	85.2 (83.8 to 86.6)	67.9 (64.9 to 70.9)
Height/waist equation	91.3 (90.3 to 92.2)	92.1 (90.1 to 94.1)	92.5 (91.4 to 93.7)	84.8 (82.8 to 86.7)
(√Height)/waist equation	90.5 (89.6 to 91.4)	93.4 (91.8 to 95.1)	91.6 (90.5 to 92.8)	82.9 (80.7 to 85.1)
Height²/(waist X √weight) equation	88.7 (87.6 to 89.7)	92.0 (90.0 to 93.9)	90.2 (89.0 to 91.5)	78.0 (75.6 to 80.3)
Height³/(waist X weight) equation	86.1 (84.9 to 87.2)	89.8 (87.7 to 91.9)	87.8 (86.5 to 89.1)	72.7 (70.1 to 75.4)
**Precision (95% CI)**				
BMI	5.4 (5.2 to 5.6)	4.3 (4.1 to 4.6)	5.2 (5.0 to 5.5)	5.6 (5.2 to 5.9)
1/BMI equation	5.1 (4.9 to 5.3)	4.4 (4.1 to 4.7)	5.0 (4.8 to 5.2)	5.5 (5.0 to 5.9)
Height/waist equation	4.1 (4.0 to 4.2)	3.7 (3.3 to 4.0)	4.0 (3.9 to 4.2)	4.1 (3.8 to 4.4)
(√Height)/waist equation	4.0 (3.8 to 4.1)	3.6 (3.3 to 3.9)	4.0 (3.8 to 4.1)	4.0 (3.7 to 4.3)
Height²/(waist X √weight) equation	4.4 (4.2 to 4.5)	3.8 (3.5 to 4.1)	4.4 (4.1 to 4.6)	4.5 (4.2 to 4.9)
Height³/(waist X weight) equation	4.6 (4.5 to 4.8)	4.0 (3.7 to 4.4)	4.6 (4.4 to 4.8)	4.9 (4.6 to 5.2)

* Values represent weighted estimates with 95% confidence intervals (95% CI) from DXA imputed data.
† Bias was calculated as the median difference between estimated and measured body fat percentage.
‡ Accuracy was calculated as the proportion of cases with <20% difference between estimated and measured body fat percentage.
§ Precision was calculated as the interquartile range of the difference between estimated and measured body fat percentage.

Supplementary Table 10. Prediction of body fat percentage in NHANES 2005-2006, by sex and ethnicity*.

	**All**	**Mexican-American**	**European-American**	**African-American**
**Women**	N=1,700	N=355	N=763	N=435
**R^2 (95% CI)**				
BMI	0.65 (0.63 to 0.67)	0.62 (0.57 to 0.68)	0.66 (0.64 to 0.69)	0.65 (0.57 to 0.72)
1/BMI equation	0.72 (0.69 to 0.74)	0.65 (0.60 to 0.71)	0.74 (0.72 to 0.77)	0.71 (0.64 to 0.78)
Height/waist equation (RFM)	0.69 (0.67 to 0.72)	0.59 (0.52 to 0.67)	0.72 (0.69 to 0.76)	0.63 (0.57 to 0.70)
(√Height)/waist equation	0.69 (0.67 to 0.72)	0.59 (0.52 to 0.66)	0.72 (0.69 to 0.75)	0.64 (0.58 to 0.69)
$Height^2$/(waist X √weight) equation	0.73 (0.71 to 0.75)	0.64 (0.58 to 0.70)	0.76 (0.73 to 0.79)	0.70 (0.63 to 0.76)
$Height^3$/(waist X weight) equation	0.73 (0.71 to 0.76)	0.64 (0.59 to 0.70)	0.76 (0.73 to 0.79)	0.70 (0.64 to 0.77)
**RMSE (95% CI)**				
BMI	4.15 (3.97 to 4.33)	3.59 (3.25 to 3.92)	4.16 (3.91 to 4.40)	4.08 (3.78 to 4.38)
1/BMI equation	3.71 (3.55 to 3.87)	3.45 (3.11 to 3.78)	3.63 (3.39 to 3.86)	3.66 (3.39 to 3.92)
Height/waist equation (RFM)	3.87 (3.72 to 4.02)	3.74 (3.38 to 4.09)	3.77 (3.54 to 3.99)	4.14 (3.86 to 4.41)
(√Height)/waist equation	3.87 (3.71 to 4.02)	3.73 (3.38 to 4.09)	3.77 (3.55 to 3.99)	4.13 (3.83 to 4.42)
$Height^2$/(waist X √weight) equation	3.63 (3.48 to 3.78)	3.50 (3.17 to 3.83)	3.52 (3.28 to 3.75)	3.78 (3.52 to 4.04)
$Height^3$/(waist X weight) equation	3.62 (3.46 to 3.78)	3.49 (3.16 to 3.82)	3.50 (3.26 to 3.75)	3.72 (3.46 to 3.98)
**Men**	N=1,756	N=395	N=811	N=423
**R^2 (95% CI)**				
BMI	0.61 (0.59 to 0.63)	0.65 (0.60 to 0.70)	0.61 (0.58 to 0.65)	0.66 (0.62 to 0.70)
1/BMI equation	0.64 (0.62 to 0.66)	0.68 (0.63 to 0.73)	0.64 (0.61 to 0.67)	0.68 (0.63 to 0.73)
Height/waist equation (RFM)	0.75 (0.72 to 0.77)	0.72 (0.67 to 0.77)	0.74 (0.71 to 0.77)	0.79 (0.76 to 0.83)
(√Height)/waist equation	0.76 (0.74 to 0.78)	0.75 (0.70 to 0.80)	0.76 (0.73 to 0.78)	0.80 (0.77 to 0.83)
$Height^2$/(waist X √weight) equation	0.72 (0.70 to 0.74)	0.72 (0.68 to 0.77)	0.72 (0.69 to 0.74)	0.76 (0.73 to 0.80)
$Height^3$/(waist X weight) equation	0.69 (0.67 to 0.71)	0.71 (0.66 to 0.75)	0.69 (0.66 to 0.71)	0.73 (0.69 to 0.77)
**RMSE (95% CI)**				
BMI	3.82 (3.70 to 3.94)	3.22 (2.95 to 3.49)	3.84 (3.69 to 3.98)	3.77 (3.52 to 4.01)
1/BMI equation	3.69 (3.57 to 3.82)	3.08 (2.84 to 3.33)	3.71 (3.56 to 3.86)	3.67 (3.39 to 3.96)
Height/waist equation (RFM)	3.10 (2.98 to 3.22)	2.89 (2.66 to 3.13)	3.15 (2.98 to 3.31)	2.96 (2.75 to 3.18)
(√Height)/waist equation	3.02 (2.91 to 3.14)	2.72 (2.49 to 2.95)	3.06 (2.91 to 3.21)	2.91 (2.69 to 3.13)

Height²/(waist X √weight) equation	3.24 (3.13 to 3.36)	2.86 (2.65 to 3.07)	3.29 (3.14 to 3.44)	3.15 (2.91 to 3.39)
Height³/(waist X weight) equation	3.42 (3.30 to 3.54)	2.95 (2.75 to 3.16)	3.47 (3.32 to 3.62)	3.37 (3.10 to 3.65)

* Values represent pooled weighted estimates with bootstrapped 95% confidence intervals (95% CI) from DXA imputed data.

Supplementary Table 11. Prediction of body fat percentage in NHANES 1999-2006, by sex and ethnicity*.

	All	Mexican-American	European-American	African-American
**Women**	N=7,961	N=1,754	N=3,870	N=1,679
**R^2 (95% CI)**				
BMI	0.63 (0.62 to 0.65)	0.60 (0.58 to 0.63)	0.65 (0.63 to 0.66)	0.65 (0.63 to 0.67)
1/BMI equation	0.71 (0.69 to 0.72)	0.65 (0.62 to 0.68)	0.72 (0.71 to 0.74)	0.73 (0.70 to 0.75)
Height/waist equation (RFM)	0.67 (0.65 to 0.68)	0.59 (0.55 to 0.63)	0.68 (0.66 to 0.70)	0.65 (0.63 to 0.68)
(√Height)/waist equation	0.67 (0.65 to 0.68)	0.59 (0.55 to 0.63)	0.68 (0.66 to 0.70)	0.65 (0.63 to 0.68)
$Height^2$/(waist X √weight) equation	0.71 (0.70 to 0.73)	0.64 (0.61 to 0.67)	0.73 (0.72 to 0.75)	0.71 (0.69 to 0.73)
$Height^3$/(waist X weight) equation	0.72 (0.70 to 0.73)	0.65 (0.61 to 0.68)	0.74 (0.72 to 0.75)	0.72 (0.69 to 0.74)
**RMSE (95% CI)**				
BMI	4.12 (4.04 to 4.20)	3.68 (3.52 to 3.84)	4.14 (4.04 to 4.25)	4.06 (3.90 to 4.22)
1/BMI equation	3.70 (3.62 to 3.78)	3.47 (3.32 to 3.63)	3.68 (3.58 to 3.78)	3.59 (3.46 to 3.72)
Height/waist equation (RFM)	3.94 (3.85 to 4.03)	3.75 (3.59 to 3.91)	3.93 (3.80 to 4.06)	4.04 (3.90 to 4.19)
(√Height)/waist equation	3.93 (3.84 to 4.03)	3.75 (3.59 to 3.90)	3.93 (3.80 to 4.06)	4.04 (3.89 to 4.19)
$Height^2$/(waist X √weight) equation	3.65 (3.56 to 3.73)	3.50 (3.35 to 3.65)	3.62 (3.50 to 3.73)	3.69 (3.56 to 3.83)
$Height^3$/(waist X weight) equation	3.62 (3.54 to 3.71)	3.48 (3.33 to 3.63)	3.59 (3.48 to 3.70)	3.65 (3.51 to 3.78)
**Men**	N=8,076	N=1,834	N=4,005	N=1,625
**R^2 (95% CI)**				
BMI	0.57 (0.56 to 0.59)	0.64 (0.61 to 0.67)	0.58 (0.56 to 0.60)	0.63 (0.61 to 0.66)
1/BMI equation	0.60 (0.58 to 0.61)	0.66 (0.63 to 0.69)	0.60 (0.58 to 0.62)	0.64 (0.61 to 0.67)
Height/waist equation (RFM)	0.75 (0.74 to 0.76)	0.72 (0.69 to 0.74)	0.75 (0.73 to 0.76)	0.79 (0.77 to 0.82)
(√Height)/waist equation	0.76 (0.74 to 0.77)	0.74 (0.72 to 0.77)	0.76 (0.74 to 0.77)	0.80 (0.78 to 0.82)
$Height^2$/(waist X √weight) equation	0.71 (0.70 to 0.73)	0.71 (0.69 to 0.74)	0.71 (0.70 to 0.73)	0.75 (0.73 to 0.78)
$Height^3$/(waist X weight) equation	0.67 (0.66 to 0.69)	0.69 (0.67 to 0.72)	0.68 (0.66 to 0.69)	0.71 (0.69 to 0.74)
**RMSE (95% CI)**				
BMI	3.99 (3.92 to 4.07)	3.25 (3.15 to 3.36)	3.93 (3.84 to 4.03)	4.08 (3.95 to 4.22)
1/BMI equation	3.88 (3.81 to 3.95)	3.17 (3.06 to 3.29)	3.81 (3.73 to 3.90)	4.03 (3.88 to 4.19)
Height/waist equation (RFM)	3.06 (3.01 to 3.12)	2.88 (2.76 to 2.99)	3.04 (2.97 to 3.11)	3.05 (2.94 to 3.17)
(√Height)/waist equation	3.02 (2.97 to 3.08)	2.76 (2.65 to 2.86)	2.99 (2.92 to 3.06)	3.04 (2.91 to 3.17)

Height²/(waist X √weight) equation	3.28 (3.22 to 3.33)	2.90 (2.79 to 3.01)	3.24 (3.17 to 3.31)	3.34 (3.21 to 3.47)
Height³/(waist X weight) equation	3.49 (3.43 to 3.55)	3.00 (2.89 to 3.11)	3.45 (3.37 to 3.52)	3.62 (3.48 to 3.76)

* Values represent pooled weighted estimates with bootstrapped 95% confidence intervals (95% CI) from DXA imputed data.

Supplementary Table 12. Prediction of body fat percentage in NHANES 1999-2006, by age category*.

	**20-39 years old**	**40-59 years old**	**≥60 years old**
**Women**	N=2,675	N=2,702	N=2,584
**R^2 (95% CI)**			
BMI	0.69 (0.67 to 0.71)	0.64 (0.62 to 0.66)	0.59 (0.56 to 0.61)
1/BMI equation	0.74 (0.73 to 0.76)	0.70 (0.68 to 0.73)	0.65 (0.63 to 0.68)
Height/waist equation (RFM)	0.70 (0.68 to 0.72)	0.65 (0.62 to 0.67)	0.51 (0.48 to 0.55)
(√Height)/waist equation	0.70 (0.67 to 0.72)	0.65 (0.62 to 0.68)	0.53 (0.49 to 0.56)
$Height^2$/(waist X √weight) equation	0.74 (0.72 to 0.76)	0.70 (0.67 to 0.72)	0.61 (0.57 to 0.64)
$Height^3$/(waist X weight) equation	0.74 (0.72 to 0.76)	0.70 (0.68 to 0.73)	0.63 (0.60 to 0.66)
**RMSE (95% CI)**			
BMI	3.97 (3.85 to 4.09)	3.96 (3.83 to 4.10)	3.66 (3.52 to 3.80)
1/BMI equation	3.61 (3.50 to 3.71)	3.57 (3.44 to 3.70)	3.35 (3.24 to 3.46)
Height/waist equation (RFM)	3.92 (3.80 to 4.04)	3.89 (3.75 to 4.04)	3.97 (3.83 to 4.11)
(√Height)/waist equation	3.93 (3.81 to 4.06)	3.88 (3.74 to 4.03)	3.91 (3.77 to 4.06)
$Height^2$/(waist X √weight) equation	3.63 (3.52 to 3.73)	3.61 (3.47 to 3.75)	3.58 (3.46 to 3.70)
$Height^3$/(waist X weight) equation	3.63 (3.52 to 3.74)	3.57 (3.44 to 3.71)	3.47 (3.36 to 3.58)
**Men**	N=2,894	N=2,655	N=2,527
**R^2 (95% CI)**			
BMI	0.63 (0.60 to 0.65)	0.58 (0.55 to 0.61)	0.52 (0.49 to 0.55)
1/BMI equation	0.64 (0.62 to 0.67)	0.59 (0.56 to 0.62)	0.52 (0.49 to 0.55)
Height/waist equation (RFM)	0.77 (0.76 to 0.79)	0.71 (0.68 to 0.73)	0.63 (0.60 to 0.66)
(√Height)/waist equation	0.78 (0.77 to 0.80)	0.72 (0.70 to 0.74)	0.63 (0.60 to 0.66)
$Height^2$/(waist X √weight) equation	0.74 (0.72 to 0.75)	0.68 (0.65 to 0.70)	0.60 (0.57 to 0.63)
$Height^3$/(waist X weight) equation	0.70 (0.68 to 0.72)	0.65 (0.62 to 0.67)	0.57 (0.54 to 0.60)
**RMSE (95% CI)**			
BMI	3.90 (3.78 to 4.02)	3.58 (3.46 to 3.70)	3.67 (3.57 to 3.77)
1/BMI equation	3.80 (3.68 to 3.92)	3.52 (3.40 to 3.64)	3.65 (3.55 to 3.75)
Height/waist equation (RFM)	3.04 (2.95 to 3.13)	2.99 (2.90 to 3.09)	3.23 (3.14 to 3.32)
(√Height)/waist equation	2.98 (2.89 to 3.07)	2.93 (2.84 to 3.03)	3.22 (3.13 to 3.31)
$Height^2$/(waist X √weight) equation	3.27 (3.17 to 3.37)	3.13 (3.03 to 3.23)	3.33 (3.24 to 3.43)
$Height^3$/(waist X weight) equation	3.50 (3.39 to 3.60)	3.29 (3.18 to 3.39)	3.46 (3.36 to 3.55)

* Values represent pooled weighted estimates with bootstrapped 95% confidence intervals (95% CI) from DXA imputed data.

Supplementary Table 13. Performance of BMI and RFM in NHANES 1999-2006.*

	All	Mexican-American	European-American	African-American
**Women**				
**Bias (95% CI)**				
BMI	−11.7 (−11.9 to −11.5)	−11.8 (−12.2 to −11.4)	−11.9 (−12.1 to −11.7)	−9.7 (−11.9 to −11.5)
RFM	0.5 (0.3 to 0.7)	1.0 (0.7 to 1.3)	0.3 (0.1 to 0.5)	1.4 (1.0 to 1.7)
**Accuracy (95% CI)**				
BMI	16.9 (15.7 to 18.0)	17.3 (14.7 to 19.9)	14.5 (13.1 to 15.8)	33.9 (31.4 to 36.4)
RFM	92.9 (92.0 to 93.7)	93.8 (92.4 to 95.3)	92.9 (91.8 to 94.1)	91.0 (89.7 to 92.3)
**Precision (95% CI)**				
BMI	5.6 (5.5 to 5.8)	5.4 (5.1 to 5.7)	5.5 (5.3 to 5.8)	5.9 (5.6 to 6.2)
RFM	5.2 (5.1 to 5.4)	4.7 (4.4 to 5.1)	5.3 (5.1 to 5.4)	5.3 (4.9 to 5.6)
**Men**				
**Bias (95% CI)**				
BMI	0.2 (0.0 to 0.3)	0.0 (−0.2 to 0.3)	−0.1 (−0.3 to 0.1)	2.6 (2.3 to 2.9)
RFM	0.1 (0.0 to 0.2)	0.6 (0.4 to 0.9)	−0.1 (−0.2 to 0.1)	0.9 (0.6 to 1.1)
**Accuracy (95% CI)**				
BMI	82.0 (80.9 to 83.0)	90.1 (88.5 to 91.6)	83.4 (82.1 to 84.6)	66.7 (64.0 to 69.4)
RFM	90.7 (89.9 to 91.5)	91.9 (90.5 to 93.3)	91.6 (90.6 to 92.6)	85.3 (83.4 to 87.2)
**Precision (95% CI)**				
BMI	5.3 (5.1 to 5.5)	4.2 (4.0 to 4.4)	5.2 (5.0 to 5.4)	5.4 (5.1 to 5.7)
RFM	4.1 (4.0 to 4.3)	3.7 (3.5 to 4.0)	4.1 (4.0 to 4.3)	4.0 (3.8 to 4.3)

* Values represent weighted estimates with bootstrapped 95% confidence intervals (95% CI) from DXA imputed data.

Supplementary Table 14. Performance of BMI, RFM and published equations for the estimation of whole-body fat percentage across age categories in NHANES 1999-2006.*

	20-39 years old	40-59 years old	≥60 years old
**Women**	N=2,675	N=2,702	N=2,584
**Bias (95% CI)**			
BMI	–10.6 (–10.8 to –10.4)	–11.7 (–11.9 to –11.4)	–13.9 (–14.1 to –13.6)
RFM	0.8 (0.5 to 1.0)	0.4 (0.2 to 0.7)	0.2 (0.0 to 0.4)
**Accuracy (95% CI)**			
BMI	19.0 (17.4 to 20.6)	18.8 (16.9 to 20.6)	9.6 (8.3 to 10.9)
RFM	91.9 (90.8 to 93.0)	93.0 (91.7 to 94.2)	94.3 (93.3 to 95.4)
**Precision (95% CI)**			
BMI	5.3 (5.1 to 5.6)	5.4 (5.1 to 5.7)	5.2 (5.0 to 5.5)
RFM	5.4 (5.2 to 5.6)	5.0 (4.7 to 5.2)	5.4 (5.1 to 5.7)
**Men**	N=2,894	N=2,655	N=2,527
**Bias (95% CI)**			
BMI	1.2 (0.0 to 1.4)	0.1 (–0.1 to 0.3)	–2.5 (–2.8 to –2.2)
RFM	0.1 (0.0 to 0.3)	0.3 (0.1 to 0.5)	–0.5 (–0.7 to –0.2)
**Accuracy (95% CI)**			
BMI	77.1 (75.5 to 78.6)	86.3 (84.8 to 87.8)	83.6 (81.9 to 85.3)
RFM	88.8 (87.4 to 90.2)	91.5 (90.2 to 92.8)	93.3 (92.2 to 94.4)
**Precision (95% CI)**			
BMI	5.2 (5.0 to 5.4)	4.8 (4.6 to 5.1)	5.1 (4.8 to 5.4)
RFM	4.1 (3.9 to 4.3)	4.0 (3.8 to 4.2)	4.3 (4.1 to 4.6)

* Values represent weighted estimates with bootstrapped 95% confidence intervals (95% CI) from DXA imputed data.

Supplementary Table 15. Model fitting of BMI and RFM for body fat percentage, fat mass and trunk fat percentage in NHANES 1999-2006*.

	**Whole-body fat percentage**	**Whole-body fat mass**	**Trunk fat percentage**
**Women (N=7,961)**			
**R^2 (95% CI)**			
BMI	0.63 (0.62 to 0.65)	0.91 (0.91 to 0.92)	0.65 (0.64 to 0.66)
RFM	0.67 (0.65 to 0.68)	0.68 (0.67 to 0.69)	0.77 (0.75 to 0.78)
**RMSE (95% CI)**			
BMI	4.12 (4.04 to 4.20)	3.70 (3.62 to 3.79)	5.10 (5.01 to 5.20)
RFM	3.94 (3.85 to 4.03)	7.13 (6.91 to 7.35)	4.17 (4.06 to 4.27)
**Men (N=8,076)**			
**R^2 (95% CI)**			
BMI	0.57 (0.56 to 0.59)	0.85 (0.84 to 0.86)	0.58 (0.56 to 0.59)
RFM	0.75 (0.74 to 0.76)	0.72 (0.71 to 0.73)	0.79 (0.78 to 0.80)
**RMSE (95% CI)**			
BMI	3.99 (3.92 to 4.07)	3.98 (3.90 to 4.07)	4.84 (4.74 to 4.94)
RFM	3.06 (3.01 to 3.12)	5.48 (5.30 to 5.66)	3.44 (3.36 to 3.51)

* Values represent pooled weighted estimates with bootstrapped 95% confidence intervals (95% CI) from DXA imputed data.

Supplementary Table 16. Comparison of diagnostic accuracy for obesity among BMI, RFM and previously published equations among participants of NHANES 1999-2006.

	**ROC - AUC**	**P value (compared with BMI)***
**Women (N=7,961)**		
BMI	0.93 (0.93 to 0.94)	
Height/waist equation (RFM)	0.93 (0.92 to 0.93)	0.007
CUN-BAE equation	0.94 (0.93 to 0.94)	0.156
Gallagher equation	0.94 (0.93 to 0.95)	<0.001
Deurenberg equation	0.93 (0.92 to 0.94)	0.567
Kagawa equation	0.92 (0.91 to 0.92)	<0.001
**Men (N=8,076)**		
BMI	0.90 (0.89 to 0.91)	
Height/waist equation (RFM)	0.95 (0.94 to 0.95)	<0.001
CUN-BAE equation	0.91 (0.90 to 0.92)	<0.001
Gallagher equation	0.91 (0.90 to 0.92)	<0.001
Deurenberg equation	0.91 (0.90 to 0.92)	0.016
Kagawa equation	0.94 (0.93 to 0.95)	<0.001

* Bonferroni-adjusted.
ROC, Receiver-operating-characteristic curve analysis; AUC, area under the curve.

References

1. National Health and Nutrition Examination Survey: Technical documentation for the 1999-2004. Dual Energy X-Ray Absorptiometry (DXA) multiple imputation data files. *Available at:* http://wwwncdcgov/nchs/data/nhanes/dxa/dxa_techdocpdf *Accessed May 17, 2016.* 2008.
2. Gallagher D, Heymsfield SB, Heo M, Jebb SA, Murgatroyd PR, Sakamoto Y. Healthy percentage body fat ranges: an approach for developing guidelines based on body mass index. *Am J Clin Nutr.* 2000;72(3):694-701.
3. Goodman-Gruen D, Barrett-Connor E. Sex differences in measures of body fat and body fat distribution in the elderly. *Am J Epidemiol.* 1996;143(9):898-906.
